# Frequencies of variants in genes associated with dyslipidemias identified in Costa Rican genomes

**DOI:** 10.3389/fgene.2023.1114774

**Published:** 2023-03-30

**Authors:** Juan Carlos Valverde-Hernández, Andrés Flores-Cruz, Gabriela Chavarría-Soley, Sandra Silva de la Fuente, Rebeca Campos-Sánchez

**Affiliations:** ^1^ Centro de Investigación en Biología Celular y Molecular, University of Costa Rica, San José, Costa Rica; ^2^ Escuela de Biología, University of Costa Rica, San José, Costa Rica

**Keywords:** dyslipidemia, genetic variant, whole genome sequences (WGS), Costa Rica, allele frequencies, pharmacogenomic, Latin America

## Abstract

Dyslipidemias are risk factors in diseases of significant importance to public health, such as atherosclerosis, a condition that contributes to the development of cardiovascular disease. Unhealthy lifestyles, the pre-existence of diseases, and the accumulation of genetic variants in some *loci* contribute to the development of dyslipidemia. The genetic causality behind these diseases has been studied primarily on populations with extensive European ancestry. Only some studies have explored this topic in Costa Rica, and none have focused on identifying variants that can alter blood lipid levels and quantifying their frequency. To fill this gap, this study focused on identifying variants in 69 genes involved in lipid metabolism using genomes from two studies in Costa Rica. We contrasted the allelic frequencies with those of groups reported in the 1000 Genomes Project and gnomAD and identified potential variants that could influence the development of dyslipidemias. In total, we detected 2,600 variants in the evaluated regions. However, after various filtering steps, we obtained 18 variants that have the potential to alter the function of 16 genes, nine variants have pharmacogenomic or protective implications, eight have high risk in Variant Effect Predictor, and eight were found in other Latin American genetic studies of lipid alterations and the development of dyslipidemia. Some of these variants have been linked to changes in blood lipid levels in other global studies and databases. In future studies, we propose to confirm at least 40 variants of interest from 23 genes in a larger cohort from Costa Rica and Latin American populations to determine their relevance regarding the genetic burden for dyslipidemia. Additionally, more complex studies should arise that include diverse clinical, environmental, and genetic data from patients and controls and functional validation of the variants.

## 1 Introduction

Dyslipidemias are a group of conditions characterized by abnormal lipid levels. High lipid profiles include hyperlipidemias or hyperlipoproteinemia. These are worldwide diseases affecting many people. In Latin American cities such as Barquisimeto, Lima, and Bogotá, this condition has been recorded in >70% of men and >50% of women (Vinueza et al., 2010). Costa Rica is no exception. In a study conducted in the 2000s involving 107,000 inhabitants of San José, it was reported that 36% of men and 22% of women had hypercholesterolemia, while 48% of men and 52% of women reported hypertriglyceridemia (Gutiérrez-Peña and Romero-Zúñiga, 2010). These conditions have been closely linked to the development of complex ailments such as cardiovascular diseases and acute pancreatitis (Bruikman et al., 2017; Pretis et al., 2018; Paredes et al., 2019), making hyperlipidemia a public health problem in the 21st century.

A sedentary lifestyle and poor eating habits can profoundly impact the development of these diseases (Brahm and Hegele, 2013). The clinical approach to these cases usually includes the implementation of exercise regimens and caloric restriction. Additionally, multiple pieces of evidence have shown that the genetic characteristics of an individual play a leading role in the development of hyperlipidemias (Johansen et al., 2011; Brahm and Hegele, 2013; Wierzbicki and Reynolds, 2019). Currently, the diseases are considered mostly polygenic. However, variants in genes such as the lipoprotein lipase (*LPL*), the low-density lipoprotein receptor (*LDLR*), and apolipoprotein B (*APOB*) tend to have more marked effects than other genes involved in lipid metabolism (Johansen et al., 2011, 2014; Lewis et al., 2015; Dron et al., 2020a, 2020b).

Most of the studies aimed at identifying the effect of the genetic component on the presence of alterations in lipid metabolism and the development of dyslipidemia have been performed mainly in Anglo-Saxon and European countries. The study by Andaleon et al. (2019) on Latin American populations is one of the most exhaustive of this kind in this region, including Central Americans. However, little is currently known in Latin American populations about the genetic variants and frequencies in genes previously linked to these conditions in other global studies.

Particularly in Costa Rica, few studies on this matter have been published. In one study, from the Dietary Fat and Heart Disease in Costa Rica project (also known as the Costa Rica Heart Study), they quantified the allelic frequencies of specific variants in the *APOC, LPL, APOE, PCSK9, FADS1-two to three*, and *USF1* genes in 4,000 individuals from the Costa Rican Central Valley. They reported an association of some of these variants with an increased risk of coronary heart disease and hyperlipidemia (Campos et al., 2001; Brown et al., 2003; Yang et al., 2004; Ruiz-Narváez et al., 2005, 2008; Gong et al., 2011; Aslibekyan et al., 2012; Yu et al., 2017). Other two research projects have focused on identifying genetic variants in regions of interest, such as the *LPL* gene and the *APOCII* promoter region in a group of 38 Costa Ricans with hypertriglyceridemia (González-Cordero, 2018; Gutiérrez-Ávila, 2019).

Here, we used data from 258 whole genomes from the Central Valley of Costa Rica to identify genetic variants in genes linked to the incidence of dyslipidemia and estimate their allelic frequencies as a proxy of genetic burden. This is the first national portrait of the frequency of previously reported risk variants in genes associated with this group of diseases obtained from genomic data. Additionally, we report the allelic frequencies of variants in genes of interest previously identified in Costa Ricans (i.e., *LDLR* and *APOCII*) and Latin American populations. The information generated in this study will help guide and contextualize future studies on dyslipidemia in Costa Rica and the region; possible next steps include validation of 40 variants of interest in a larger population and determining the impact of these findings on the national healthcare system. Moreover, this study reflects the importance of studies that include clinical, environmental, and genetic data from patients and controls.

## 2 Materials and methods

### 2.1 Samples and genomic data

We used anonymized whole genome sequence data from two collections. One is from the repository PSYCH-CV, a collection of Costa Rican WGS from the NIMH-funded (National Institute of Mental Health) study U01MH105630-04S1, which included subjects with mania and psychosis and their relatives recruited under different studies and anonymized in the WGS data repository (Chavarria-Soley et al., 2021). We selected only unrelated individuals without a mental disorder diagnosis from the families, for a total of 23 individuals. The sequencing was carried out using the Illumina HISEQ 2000 with paired ends. The data had a minimum coverage of 30x and a read length of 100 pb. The data were previously aligned with the BWA-MEM tool of the BWA V0.7.15 package using the GRCH38 reference genome and stored in CRAM format.

The second data set was from the project The Genetic Epidemiology of Asthma in Costa Rica (dbGAP phs000988.V4.P1). Individuals without a family relationship and an asthma diagnosis were selected using the dbGAP metadata. In total, 234 subjects met these criteria (called dbGAP-CV, Supplementary Table S1), and CRAM files were downloaded from the database. The genomes of both databases were added to a single group of 258 subjects called CR-WGS for the variant annotation.

### 2.2 Variant discovery and genotype

The analysis was limited to all coordinates corresponding to the transcriptome according to the GFF3 of Ensembl 106 for the GRCh38 genome, including miRNAs and lncRNAs. We call these regions the exome. Additionally, we extracted two sets of ancestry informative markers (AIMs) sets reported by Campos-Sánchez et al. (2013) and by Galanter et al. (2012). Each coordinate interval was extended to 300 bp upstream and downstream (Table 1).

**TABLE 1:** Genomic coordinates selected for variant calling.

Use in the study	Identifier	Source of coordinates	Source of identifiers
Quality control analysis	RNA coding regions from *Ensembl Release 106*		
Variant training set for GATK, ‘*Variant Quality Score Recalibration’* (VQSR)	RNA coding regions from *Ensembl Release 106*		
Ancestry estimates based on Costa Ricans studies	78 variants from dbSNP	dbSNP variants: *Ensembl Genes 106* database, GRCh38.p13.genome coordinates extracted from *BioMart*	Campos-Sánchez et al. (2013)
Ancestry estimates compared to American groups from *1KGP phase 3*	446 variants from dbSNP	dbSNP variants: *Ensembl Genes 106* database, GRCh38.p13. genome coordinates extracted from *BioMart*	Galanter et al. (2012)
Exonic variants in genes involved in lipid metabolism and dyslipidemias	*ABCA1, ABCG1, ABCG4, ABCG5, ABCG8, ABHD5, ANGPTL3, APOA1, APOA2, APOA4, APOA5, APOB, APOC1, APOC2, APOC3, APOC4, APOD, APOE, APOF, APOH, APOL1, APOL2, APOL3, APOL4, APOL5, APOL6, APOM, APOO, CD36, CELSR2, CETP, CILP2, CREB3L3, CYP26A1, FADS1, FADS2, FADS3, GALNT2, GCKR, GPD1, GPIHBP1, HMGCR, KLHL8, LCAT, LDLR, LDLRAP1, LIPA, LIPC, LIPE, LIPG, LMF1, LPL, LRP1, MLXIPL, MTTP, MYLIP, NCAN, NPC1L1, PCSK9, PLA2G7, PLIN1, PLTP, PNPLA2, PPARA, SCARB1, SORT1, STAP1, TRIB1, USF1*	Genetic symbols: *Ensembl Genes 106* database, GRCh38.p13. genome coordinates extracted from *BioMart*	Plaisier et al. (2009), Nakayama et al. (2010), Johansen et al. (2011, 2014), Johansen and Hegele (2011), Vasquez-Vidal (2014), Lewis et al. (2015), Dron et al. (2019, 2020b), Sarraju and Knowles (2019)

As a quality control measure on the reads, duplicate reads were first removed using the MarkDuplicates tool, which is part of the GATK package. Next, to adjust for observed systematic errors caused by the sequencer, the GATK machine learning model called Base Quality Score Recalibrator was implemented using the BaseRecalibrator and ApplyBQSR commands.

We used HaplotypeCaller, GenomicsDBImport, GenotypeGVCF, and MergeVcfs for indel-like or SNV-like variant calling. During this process, tGRCh38/hg38 was selected as the reference genome and the dbSNP Build 151 variant database was used as the reference source for variants.

As a quality check on the identified variants, an error score referred to as VQSLOD was calculated for the identified variants using GATK’s machine learning model, Variant Quality Score Recalibrator (VQSR). To do this, metrics obtained for each variant are fed to the VQSR model, including variant depth, strand bias, and quality of the variant assigned in the previous stage, along with lists of variants with different degrees of confidence (DePristo et al., 2011). The evaluation of variant calling errors was performed for indels and SNVs separately.

The databases supplied to the VQSR model are stored in GATK’s repository “Resource bundle” “genomics-public-data”, except for the dbSNP v151 database, which was extracted from the FTP site of the National Center for Biotechnology Information of the United States (NCBI). To calculate the error score in the indels, those highly validated in the Mills and 1,000 genomes gold standard data set (Mills et al., 2006) were considered true variants. The training data were the genotypes from the first phase of the 1000 Genomes Project (1KGP) study obtained with the Axiom Exome Plus chip. The dbSNP v151 database was also supplied to the model, but it was considered a database with a lower degree of validation.

**FIGURE 1:**
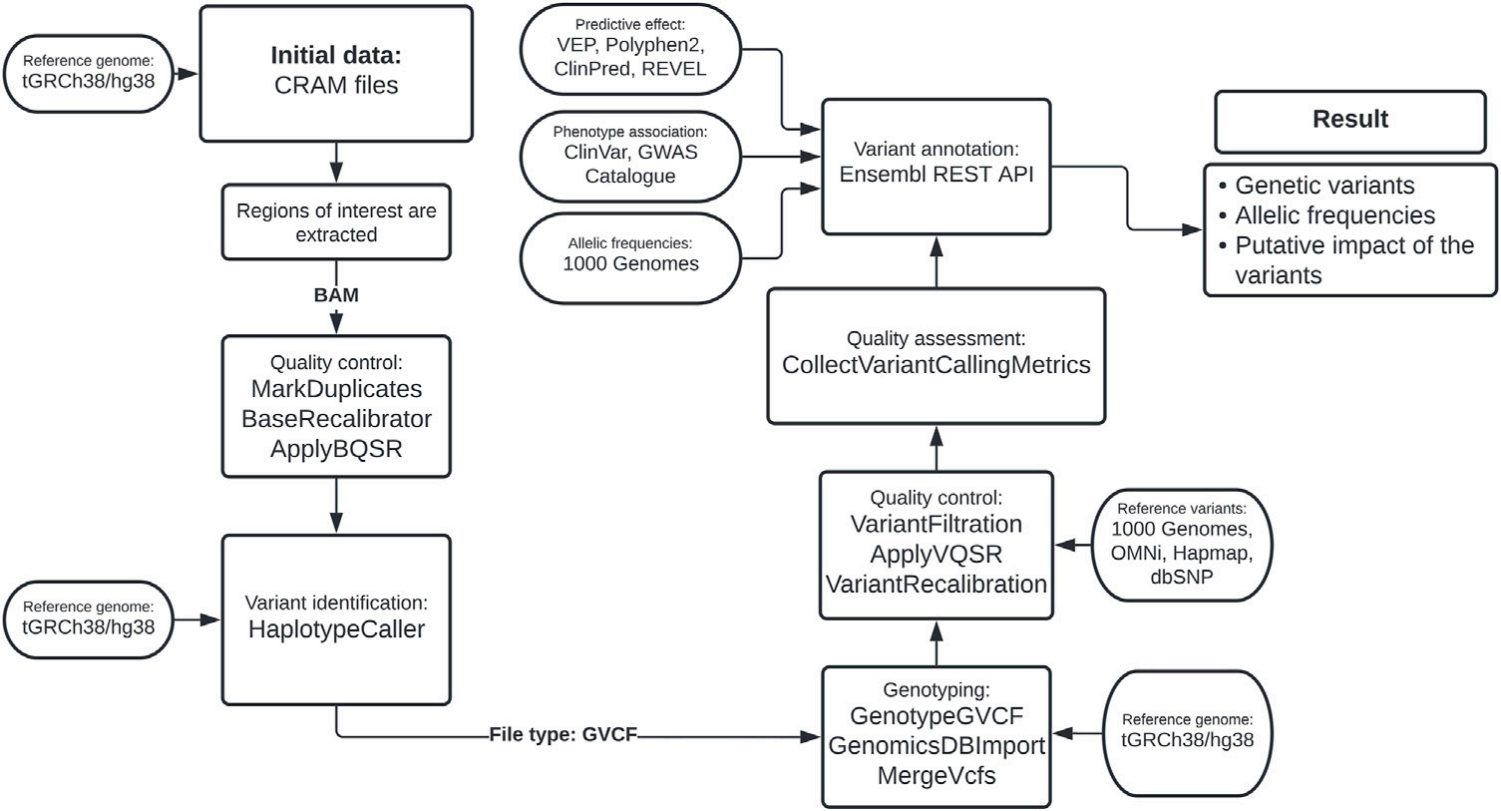
Bioinformatics pipeline based on the Best Practices Variant Calling from GATK.

To calculate error scores for SNVs, we considered true variants as those found in the HapMap database phase 3 release 3, part of the International HapMap Project (Consortium et al., 2010). The training databases were defined as the panel of phase 3 1KGP genotyped with the OMNI 2.5 chip and the database of genotypes with a high confidence level from phase 1 of 1KGP. Finally, the dbSNP database was the reference source for known variants. Using ApplyVQSR, we excluded from further analysis variants with a VQSLOD of less than 97.5% of SNVs-like variants and 95% of indel-like variants. This bioinformatics pipeline is summarized in Figure 1.

### 2.3 Evaluation of bioinformatics processing

Using the GATK CollectVariantCallingMetrics tool, the transition vs. transversion ratio (Ti/Tv) and the heterozygous vs. homozygous alternative allele ratio (Het/non-ref Hom) were calculated, metrics commonly used to describe the quality of the variant calling process. These metrics were obtained separately for each chromosome and at the exome level. The values ​​obtained were compared between both Costa Rican cohorts using a t-test.

Additionally, to evaluate the concordance between the allele frequencies, a linear model was generated to contrast the frequencies previously reported in the Costa Rica Heart Study publications and those obtained for CR-WGS (Brown et al., 2003; Ruiz-Narváez et al., 2005; Ruiz-Narvaez et al., 2010).

### 2.4 Genetic ancestry analysis

To determine if the subjects included in both Costa Rican cohorts present an ancestry profile that fits within the pattern observed in other Latin American populations, we used the genotypes of 446 AIMs (Ancestry Informative Markers) described by Galanter et al. (2012), and the ancestral populations from 1KGP panel (European-EUR, African-AFR, and East Asian-EAS) (Auton et al., 2015; Sudmant et al., 2015). We used the EAS group as a proxy of Native American ancestry since most of the ancestry of Native Americans comes from the East Asian population (Wang et al., 2019), given the scarcity of genomic data for this population group. Subjects from Barbados (ACB) and subjects with African ancestry from the South West of USA (ASW) were not considered members of AFR, nor were Utahns (CEUs) part of the EUR group since they are Americans. The CLM (Colombia), MXL (Mexico), PEL (Peru), and PUR (Puerto Rico) groups were considered Latin American.

The genotypes of the 446 AIMs were downloaded for 200 randomly selected individuals for each ancestral group (AFR, EUR, EAS) and all available samples for ACB, ASW, CEU, CLM, MXL, PEL, and PUR individuals. Genotypes were extracted for both Costa Rican cohorts, which were integrated with the 1KGP dataset. Principal component analysis (PCA) was performed using the number of alternative alleles by AIM. Only AIMs without missing genotypes were included. We estimated the similarity relationships between American populations and AFR, EUR, and EAS using the allelic frequencies in the TreeMix v1.13 program (Pickrell and Pritchard, 2012).

To assess whether the ancestry of both Costa Rican cohorts was consistent with the profile previously reported for subjects from the Costa Rican Central Valley, we performed a genetic admixture analysis using STRUCTURE v2.3.4 (Hubisz et al., 2009) using 78 AIMs described by Campos-Sánchez et al. (2013). We used the same ancestral groups as before (AFR, EUR, EAS). We integrated the genotypes for such AIMs in both Costa Rican cohorts and those reported for Costa Rican groups from the North Region (2013-NR), South Region (2013-SR), the Caribbean region (2013-CR), and the Ventral Valley (2013-CV) (Campos-Sánchez et al., 2013). The integrated database contained 1,067 individuals for the analysis in STRUCTURE (Hubisz et al., 2009). The run parameters were: ‘Length of Burnin Period’ or the number of iterations to reduce the effect of the initial configuration set to 50,000, ‘Number of MCMC Reps after Burnin’ or the number of iterations of the model to obtain accurate estimates set to 100,000, genetically admixed individuals, the groups could have correlated allele frequencies, and the ancestral groups were EUR, AFR and EAS groups. With these parameters, we performed ten simulations assuming that the population had three ancestral groups. These results were merged using CLUMPP and DISTRUCT through the CLUMPAK tool (Rosenberg, 2004; Jakobsson and Rosenberg, 2007; Kopelman et al., 2015). Three plots were generated, one representing genetic structure, a ternary plot of genetic admixture, and a principal component analysis (PCA) using the number of alternative alleles per variant. Only AIMs with complete genotypes were included. Kruskal-Wallis test was applied to determine ancestry similarities among Costa Rican and Latin American populations, from there we built 95% confidence intervals considering Tukey correction to identify specific differences between pairs of populations.

### 2.5 Annotation of variants

We studied the variants identified within a set of 69 genes that have a key role in lipid metabolism or that contain variants that have been associated with changes in blood lipid levels (Table 1). We annotated the variants found in the regions of interest with information hosted in Ensembl release 109 using its REST API v15.5 (Cunningham et al., 2021). Pathogenicity predictions, phenotypic associations, and population genetics information were extracted for each variant.

The variant type was determined using Variant Effect Predictor (VEP) v7 (Cunningham et al., 2021). *In silico* predictions of pathogenicity for missense variants were generated using the traditional tools PolyPhen2 and SIFT (Flanagan et al., 2010) and two more recently developed tools, ClinPred and REVEL (Ioannidis et al., 2016; Alirezaie et al., 2018; Gunning et al., 2021). Phenotypic association annotations were done with Ensembl API REST which uses ClinVar and NHGRI-EBI GWAS catalog databases (Landrum et al., 2017; Buniello et al., 2019).

To contrast the variant´s population frequencies found in the CR-WGS group with those reported in extensively characterized populations, we collected the frequencies of the 1KGP, EAS, EUR, AFR, AMR, and all 1KGP (ALL) groups. Fisher’s exact tests were performed to determine which of the variants found have a different allelic frequency in the group of Costa Rican genomes compared to the 1KGP populations. A significance level of 0.05 adjusted with the Bonferroni correction was used as the threshold to determine if the frequency between the two populations was different.

### 2.6 Identification and characterization of variants of interest

The study considered a polymorphic site as a variant of interest if (1) it was a risk variant according to three or more sources of functional annotation or if (2) the variant was previously reported in Costa Rica or Latin America within the context of metabolism of lipids and dyslipidemias. This produced two lists of variants of interest: one consisted of risk variants annotated by bioinformatic predictions found in the genes from Table 1, and the other includes the variants that have been reported in Costa Ricans and Latin Americans in the genes of interest in the context of lipid metabolism or dyslipidemia.

The list of risk variants with more than one count determined by bioinformatic predictions met at least three of the following criteria: (1) be categorized by PolyPhen2 as possibly harmful (P) or probably harmful (D), (2) being categorized by SiFT as a deleterious variant by having a score less than 0.05, (3) having an index calculated by REVEL greater than 0.5 (it groups 13 predictive tools), (4) having the ClinPred score greater than 0.5 or (5) having a phenotype reported by ClinVar or NHGRI-EBI GWAS catalog which was related to lipid metabolism or an increased risk of developing and suffering from dyslipidemia. The pharmacogenomics variants were identified from ClinVar and NHGRI-EBI GWAS catalog and annotated with PharmGKB (www.pharmgkb.org).

We used the jVenn tool (Bardou et al., 2014) to generate Venn diagrams to visualize the consensus between the different sources in determining risk variants.

We calculated the number of variants in homozygous and heterozygous states, and the total present per subject to reflect the genetic burden of dyslipidemia-related variants in the population. These metrics were obtained for the set of variants categorized by VEP as LOW, MODERATE, and HIGH risk, and the set of variants categorized as variants of interest in the present study. The data was represented in distribution plots.

### 2.7 Code for bioinformatic analysis

In addition to the tools mentioned above, we used the free programming languages Python 3.7 and R 4.1.2. Python was used to manage the variant call workflow, annotate the variants, manipulate the data, and generate visualizations. R was used to generate the visualizations produced from the TreeMix results. All code can be found in the GitHub repository https://github.com/jcvalverdehernandez/cr_dislipidemia_2022.

## 3 Results

### 3.1 Variant call metrics met exome quality standards

**FIGURE 2:**
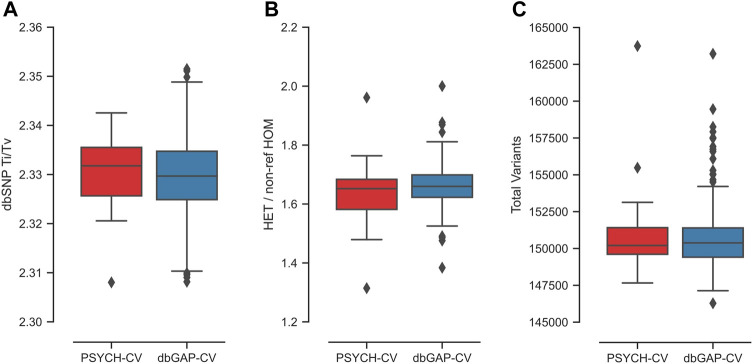
Exome quality metrics for the variant calling process performed in the PSYCH-CV and dbGAP-CV cohorts. **(A)** T_I_/T_V_ ratio per individual calculated from variants reported in dbSNP, **(B)** HET/non-ref HOM ratio per individual, **(C)** number of variants identified per individual.

The relationship Ti/Tv obtained for both datasets had a mean of 2.33 (Figure 2A). For exomes, it is reported that Ti/Tv values around 3.0 usually indicate that the data have adequate quality (Wang et al., 2015). This metric is sensitive to the genome region and functionality; thus, including intronic regions could reduce this ratio, similar to what we observe in our data. We used transcriptome coordinates that include coding and non-coding sequences (miRNAs and lncRNAs), as specified in the transcript coordinates from Ensembl 106.

The average HET/non-ref HOM ratio observed for both cohorts was 1.66 (Figure 2B). The expected value of this index is 2.0 for whole-genome sequencing variants. However, this highly depends on ancestry (Wang et al., 2015). In the study by Wang et al. (2015), average exome estimates varied from 1.4 to two in Asians and Africans, respectively.

Additionally, an exome average of 137,593 SNVs and 13,273 indels were identified per individual for both cohorts (Figure 2C). All metrics per chromosome and cohort are in Supplementary Figure S1; Supplementary Table S1. Moreover, PSYCH-CV and dbGAP-CV presented similar metrics for the three metrics (t-test *p*-value >0.05).

Finally, allelic frequencies previously reported at various polymorphic sites in the Costa Rica Heart Study were significantly correlated (r = 1.00, *p* = 1.8e-13) with those observed in CR-WGS. This result suggests a high similarity between these cohorts and that variant calling was accurate (Supplementary Figure S2).

### 3.2 The ancestry of Costa Rican genomes is consistent with previous studies

The ancestry analyses validated that PSYCH-CV and dbGAP-CV cohorts have a genetic profile consistent with that expected from a random sample of Costa Ricans from the Central Valley. They also reveal an ancestry profile similar to other Latin American groups in 1KGP, such as CLM, MXL, and PEL.

**FIGURE 3:**
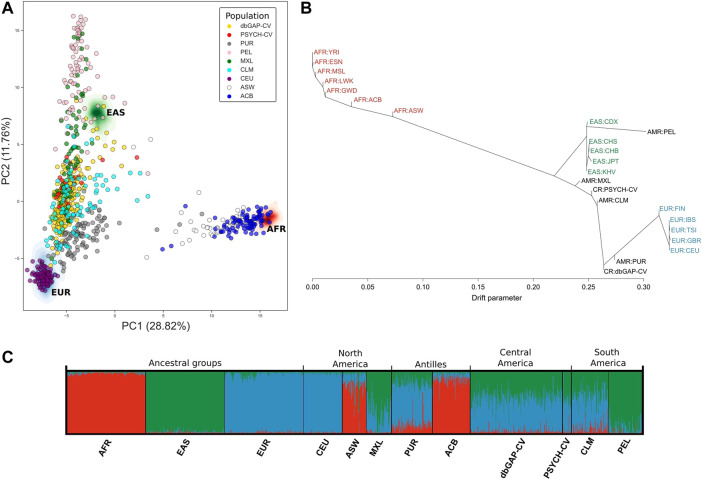
Genetic similarity between Latin American individuals based on genotypes of 446 AIMs reported by Galanter et al. (2012). **(A)** Principal component analysis. **(B)** Genetic relationships between the populations included in the analysis according to TreeMix estimates. **(C)** Individual genetic structure plot. Featured 1KGP populations - EUR: Eastern Europe, AFR: Africa, EAS: Eastern Asia, ACB: Barbados, ASW: African Ancestry in Southwest US, CEU: Utah, CLM: Colombia, MXL: Mexico, PEL: Peru, PUR: Puerto Rico, PSYCH-CV: Psychiatric study Central Valley, dbGAP-CV: dbGAP Central Valley.

Principal component analysis (PCA) captured around 40.58% (between principal components 1 and 2) of the genetic variation using the panel of 446 AIMs in the three ancestral groups and the six American groups (Figure 3A). We observed that the PSYCH-CV and dbGAP-CV individuals appear to have more similarity with the Colombian (CLM) subjects in European and Asian ancestry, and in the AFR only for PSYCH-CV. Additionally, PSYCH-CV presented similarities with the AFR and EAS component of Mexicans (MXL) (Supplementary Table S3). These observations were verified by building 95% confidence intervals (Supplementary Table S4), which are also reflected in the genetic structure plot (Figure 3C). The genetic distance tree also groups Costa Rican genomes with Latin American and European groups (Figure 3B).

**FIGURE 4:**
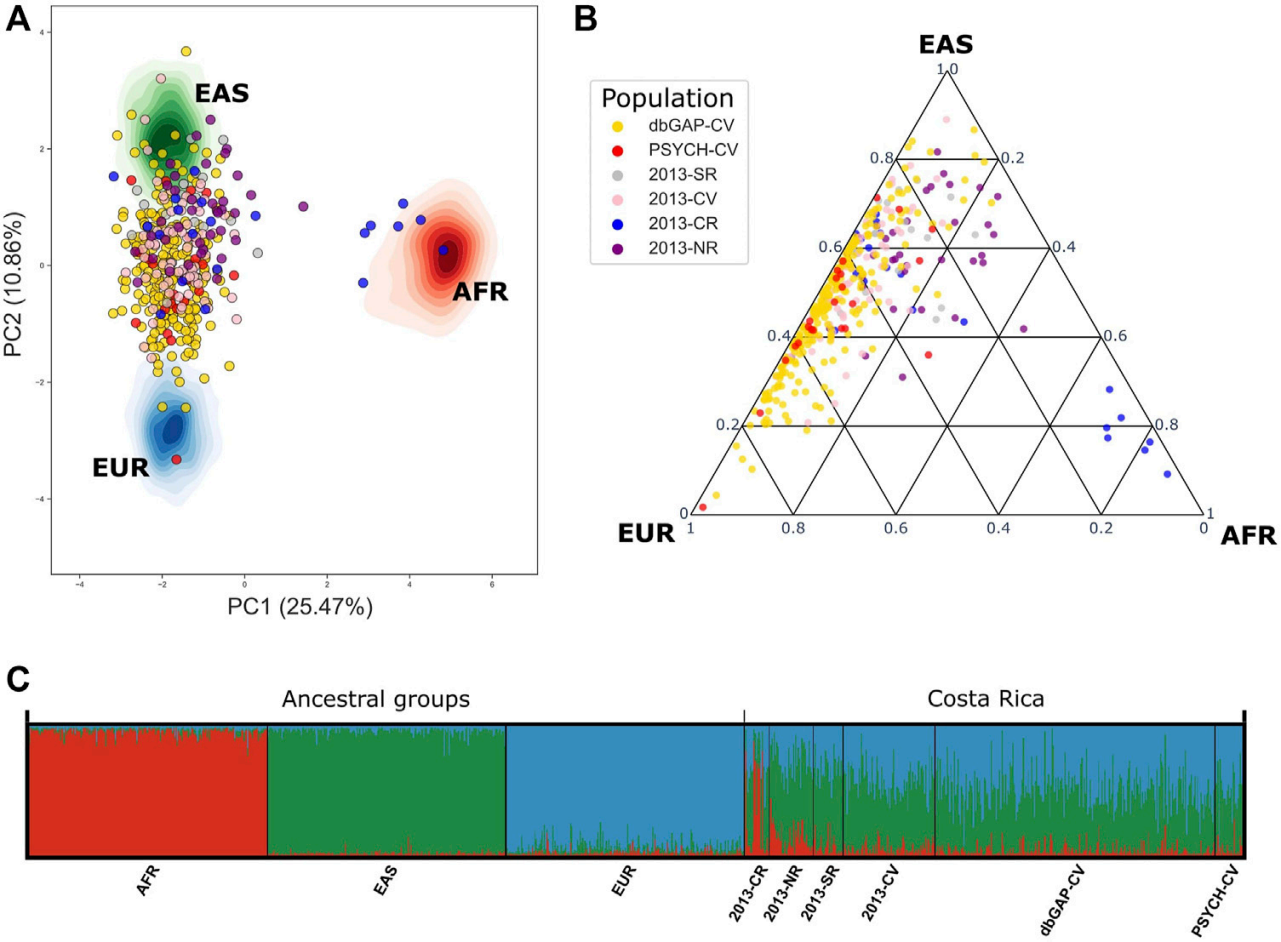
Genetic admixture in PSYCH-CV and dbGAP-CV using 78 AIMs reported by Campos-Sánchez et al. (2013). **(A)** Principal component analysis, **(B)** Genetic admixture ternary diagram. **(C)** Individual genetic structure plot. AFR: Africa, EAS: East Asia, EUR: Europe, AMR: Latin America, 2013-CR: Costa Ricans from the Caribbean Region, 2013-NR: Costa Ricans from the North Zone, 2013-SR: Costa Ricans from the South Zone, 2013-CV: Costa Ricans from the Central Valley, PSYCH-CV: Psychiatric study Central Valley, dbGAP-CV: dbGAP Central Valley.

When contrasting the genetic ancestry of PSYCH-CV and dbGAP-CV using 78 AIMS we observed complete similarity in all three ancestry components among them. Using these same markers we compared ancestry with the Costa Rican groups described by Campos-Sánchez et al. (2013) and observed the most significant similarity with the Central Valley group (2013-CV) in all three ancestry components for PSYCH-CV, but only for AFR and EAS for dbGAP-CV. Moreover, both groups showed similar AFR ancestry compared to the South (2013-SR), and EAS ancestry compared to the Caribbean Region (2013-CR). PSYCH-CV also presented AFR ancestry similar to 2013-CR (Figures 4A, B; Supplementary Table S3). These observations were verified by building 95% confidence intervals (Supplementary Table S4). The rest of the confidence intervals reflected statistically significant differences. The PCA captured approximately 36.33% of the genetic variation between principal components 1 and 2. These results provided confidence that CR-WGS represented the Central Valley population of Costa Rica.

### 3.3 Polymorphic sites identified in genes of interest

We identified 2,600 polymorphic sites in CR-WGS in the 69 genes of interest (Table 1) consisting of 2,460 SNVs and 140 indels (Table 2). However, only 2,553 were annotated in dbSNP. We detected 47 new variants not reported previously in dbSNP. Multiallelic variants represented 2.9% of all variants detected.

**TABLE 2:** Variant calling statistics for the panel of 69 genes involved in lipid metabolism.

Metric	Total	SNVs	Indels
Variants identified	2,600	2,460	140
Not in dbSNP	47	44	3
In dbSNP	2,553	2,416	137
Multiallelic	75	37	38
Biallelic	2,525	2,423	102

**FIGURE 5:**
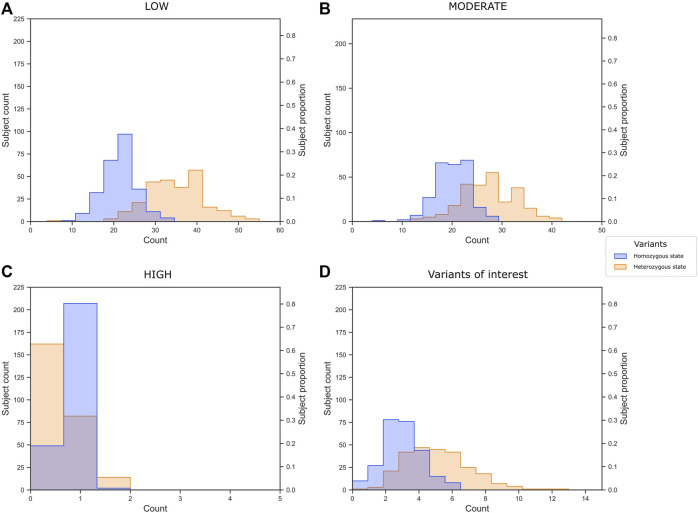
Variant burden of **(A)** low, **(B)** moderate, and **(C)** high impact variants annotated by VEP, and **(D)** the 40 variants of interest selected in the present study.

We classified 2,277 variants (unique rsIDs) into 2,769 impact annotations assigned in VEP based on the *in silico* consequence of the variant according to the Sequence Ontology (SO) term. This means that a variant could have different impact annotations depending on the region of the gene and the alternative transcript they belong to. For example, the rs5088 in *APOA2* had five annotations: intron variant, synonymous variant, 3-prime UTR variant, downstream gene variant, and splice region variant; three had a MODIFIER, and two had a LOW impact. In summary, 349 variants had a LOW impact (low risk of affecting gene transcripts), 397 MODERATE, and eight HIGH risks. It was impossible to assign an expected risk to consequences assigned to 1,941 of the variants using VEP; these consequences are referred to as MODIFIER (Supplementary Table S3). To get an idea about the genetic burden for dyslipidemia in our sample, we plotted the number of variants per individual (Figures 5A–C). The subjects presented on average 56.22 LOW impact variants (34.9 and 21.36 in heterozygous and homozygous state, respectively), 47.29 MODERATE impact variants (27.23 and 20.06 in heterozygous and homozygous state, respectively), and 1.03 HIGH impact variants (0.82 and 0.43 in heterozygous and homozygous state, respectively).

**FIGURE 6:**
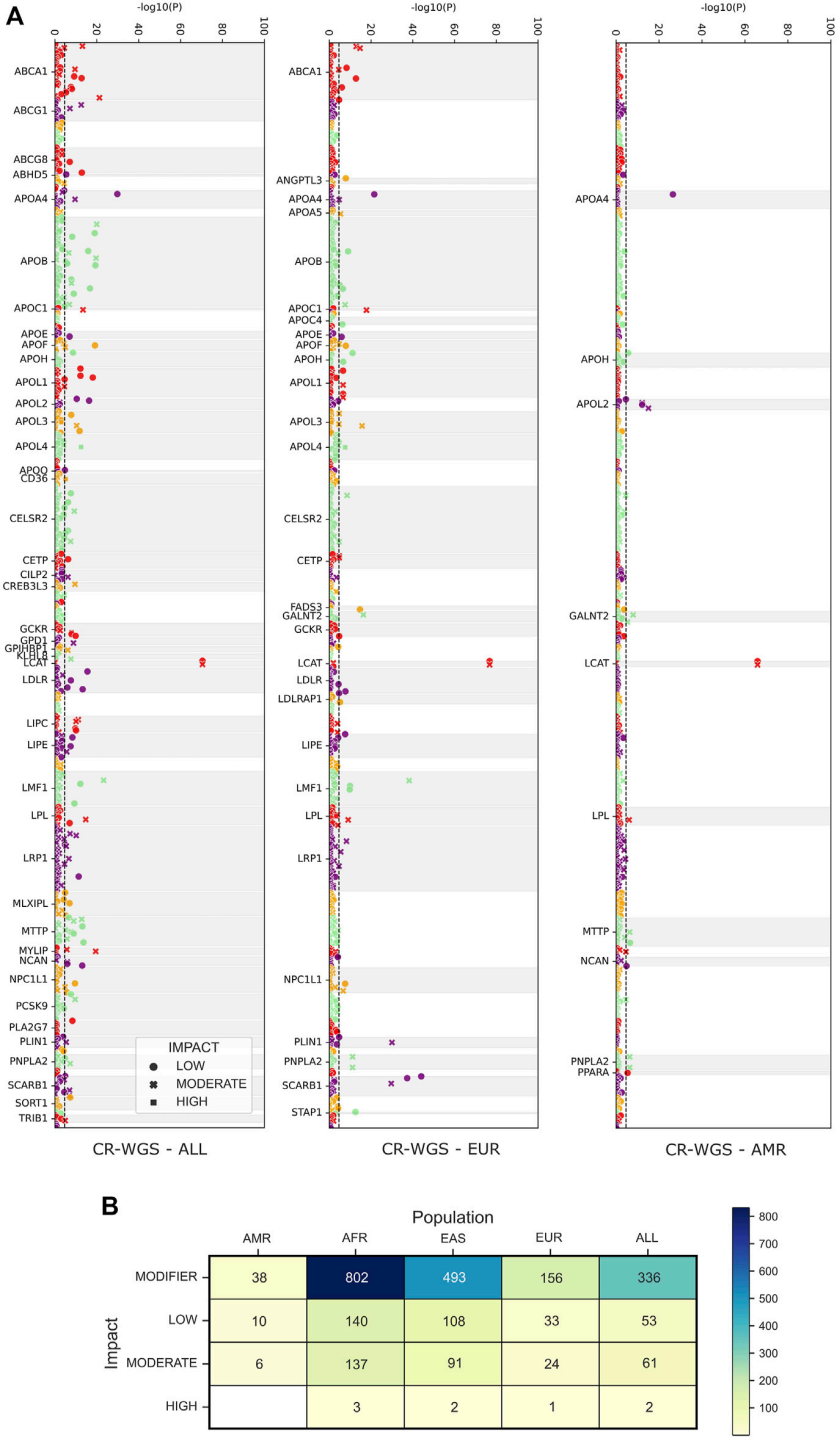
Observed differences between allelic frequencies in genes associated with lipid metabolism in Costa Ricans compared to those reported in 1KGP. **(A)** Probability, according to Fisher’s test, that the polymorphic sites have differences in their allele frequencies. The dotted line marks the significance threshold with the Bonferroni fit. Variants are categorized as LOW, MODERATE, and HIGH by VEP. **(B)** The number of variants with allelic frequencies significantly different from those observed in the Costa Rican cohort studied. CR-WGS: Costa Rican genomes evaluated in this study, ALL: all Subjects from 1KGP phase 3, EAS: East Asia, EUR: Europe, AFR: Africa, AMR: Latin America.

According to Fisher’s exact tests implemented to contrast the allele frequencies of the 2,174 variants detected in CR-WGS and those of the groups belonging to 1KGP, we observed that AMR, EUR, and ALL groups are the most similar to CR-WGS (Figure 6A). These differed individually from CR-WGS in 54, 214, and 452 allelic frequency variants, respectively (Figure 6B). On the other hand, EAS and AFR presented statistically significant differences in the frequency of the alleles of 694 and 1,082 polymorphic sites compared to CR-WGS, respectively (Supplementary Figure S4).

The eight variants associated with high-risk consequences according to VEP are summarized in Table 3. These are located in eight genes and include stop gained and start lost annotations; most were heterozygous and presented 1 to 37 copies in CR-WGS. Interestingly, rs328G and rs132642T are homozygous in two different individuals each. SNV rs328 was reported as benign in other Latin American studies and ClinVar (Table 6), while rs132642 has no annotation in ClinVar. Allele frequencies from 1KGP and gnomAD exomes are low (up to 11%, Table 3).

**TABLE 3:** High-risk variants frequency and presence of homozygous individuals for the alternate allele in CR-WGS.

Gene	dbSNP rsID	Alleles (REF/ALT)	Impact	Alternative allele frequency in CR-WGS (count)	Samples homozygous for least frequent allele	Depth of REF:ALT in least frequent allele	1KGP frequency Global for least frequent allele	gnomAD exomes frequency Global for least frequent allele
APOC4	rs5164	G/A	stop_gained	0.0019 (1)			0.0027	0.0004
APOL3	rs132642	T/A	start_lost	0.9027 (464)	2	0:30, 0:37	0.0584	0.1146
APOL4	rs192225524	C/A	stop_gained	0.0311 (16)			0.0009	0.0005
CD36	rs3211938	T/G	stop_gained	0.0019 (1)			0.0309	0.0061
GCKR	rs146053779	C/T	stop_gained	0.0096 (5)			0.0014	0.0009
GPD1	rs144009925	A/G	start_lost	0.0039 (2)			-	0.0003
LPL	rs328	C/G	stop_gained	0.0719 (37)	2	0:27, 1:34	0.0924	0.0921
SCARB1	rs749801989	T/C	start_lost	0.0116 (6)			-	0.0001

**FIGURE 7:**
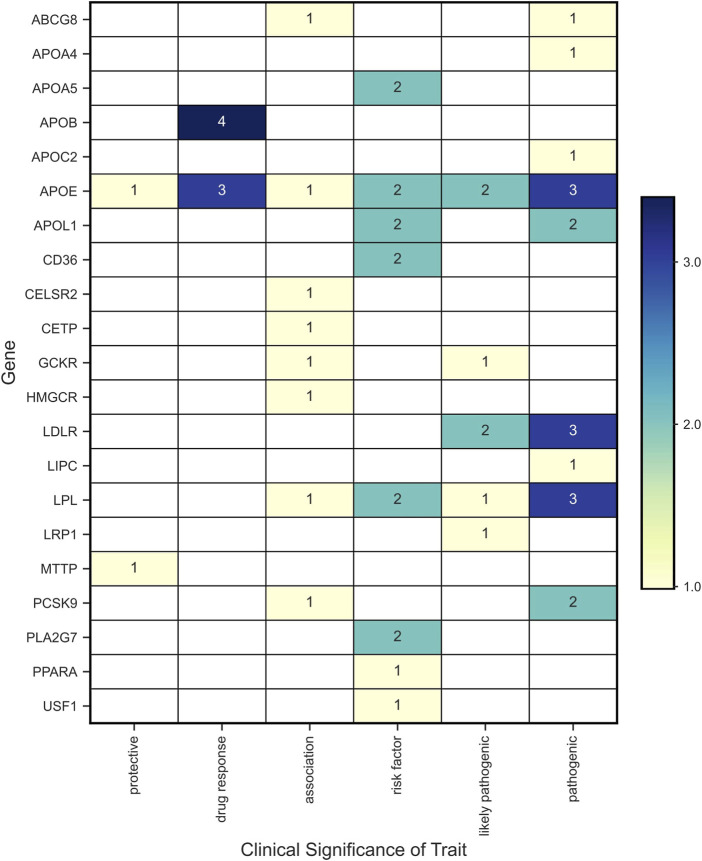
Variants with clinical significance according to the phenotypic associations reported in ClinVar.

Forty-one variants in 21 genes were associated with phenotypic traits categorized as protective, drug response, association, risk factor, likely pathogenic, and pathogenic (Figure 7). The genes with more than one variant with phenotypic traits categorized as risk or pathogenic factors (i.e., risk factor, pathogenic or likely pathogenic) were *APOA5, APOB, APOE, APOL1, CD36, GCKR, LDLR, LPL, PCSK9,* and *PLA2G7*.

Seven variants were annotated with features associated with drug response and two with protective features in *APOB, APOE*, and *HMGCR* genes (Table 4). The allelic frequencies of the alternate allele ranged from 0.01 to 0.76. These nine variants are present in 1KGP populations but we observed statistical differences in the allelic frequencies of seven of the variants. All variants presented annotations in ClinVar, including associations with traits such as warfarin, atorvastatin, and statins responses, and one protective against metabolic syndrome.

**TABLE 4:** Variants found in genes of interest that are associated phenotypically with pharmacogenomic or protective traits against diseases. CR-WGS: Costa Rican genomes evaluated in this study, ALL: all Subjects from 1KGP phase 3, EAS: East Asia, EUR: Europe, AFR: Africa, AMR: Latin America. * Significantly different allelic frequency (*p* < 0.05) compared to CR-WGS.

**Gene**	**dbSNP rsID**	**Alleles (REF/ALT)**	**Alternative allele frequency**						**Protective or pharmacogenetic traits**
			**CR-WGS**	**1KGP Phase 3**					
				**ALL**	**EUR**	**EAS**	**AFR**	**AMR**	
APOB	rs1042034	C/T	0.76163	0.62959*	0.78230	0.27976*	0.87594*	0.74927	**Allele T per ClinVar:** *Warfarin response*
APOB	rs1367117	G/A	0.34496	0.16932*	0.29821	0.11507*	0.07791*	0.28674	**Allele A per ClinVar:** *Warfarin response* **Allele A per HGRI-EBI GWAS catalog:** *Medication use HMG CoA reductase inhibitors*
APOB	rs679899	G/A	0.40116	0.48502	0.47415	0.86408*	0.13010*	0.39193	**Allele A per ClinVar:** *Warfarin response*
APOB	rs693	G/A	0.44961	0.25099*	0.44234	0.06150*	0.20953*	0.37752	**Allele A per ClinVar:** *Warfarin response*
MTTP	rs3816873	T/C	0.27432	0.24980	0.26043	0.13591*	0.26096	0.17867	**Allele C per ClinVar:** *Metabolic syndrome, potection against*
APOE	rs429358	T/C	0.07004	0.15055*	0.15506*	0.08630	0.26777*	0.10374	**Allele C per ClinVar:** *Warfarin response*
APOE	rs7412	C/T	0.06615	0.07507	0.06262	0.10019	0.10287	0.04755	**Allele T per ClinVar:** *atorvastatin response - Efficacy, Warfarin response* **Allele T per NHGRI-EBI GWAS catalog:** *Response to statins (LDL cholesterol change), Lipoprotein-associated phospholipase A2 activity change in response to darapladib treatment in cardiovascular disease*
APOE	rs769450	G/A	0.31712	0.32727	0.41153	0.21825	0.35022	0.29682	**Allele A per ClinVar:** *Warfarin response*
HMGCR	rs17238540	T/G	0.01362	0.03554	0.01689	-	0.10816*	0.02449	**Allele G per ClinVar:** *Statins, attenuated cholesterol lowering by*

**FIGURE 8:**
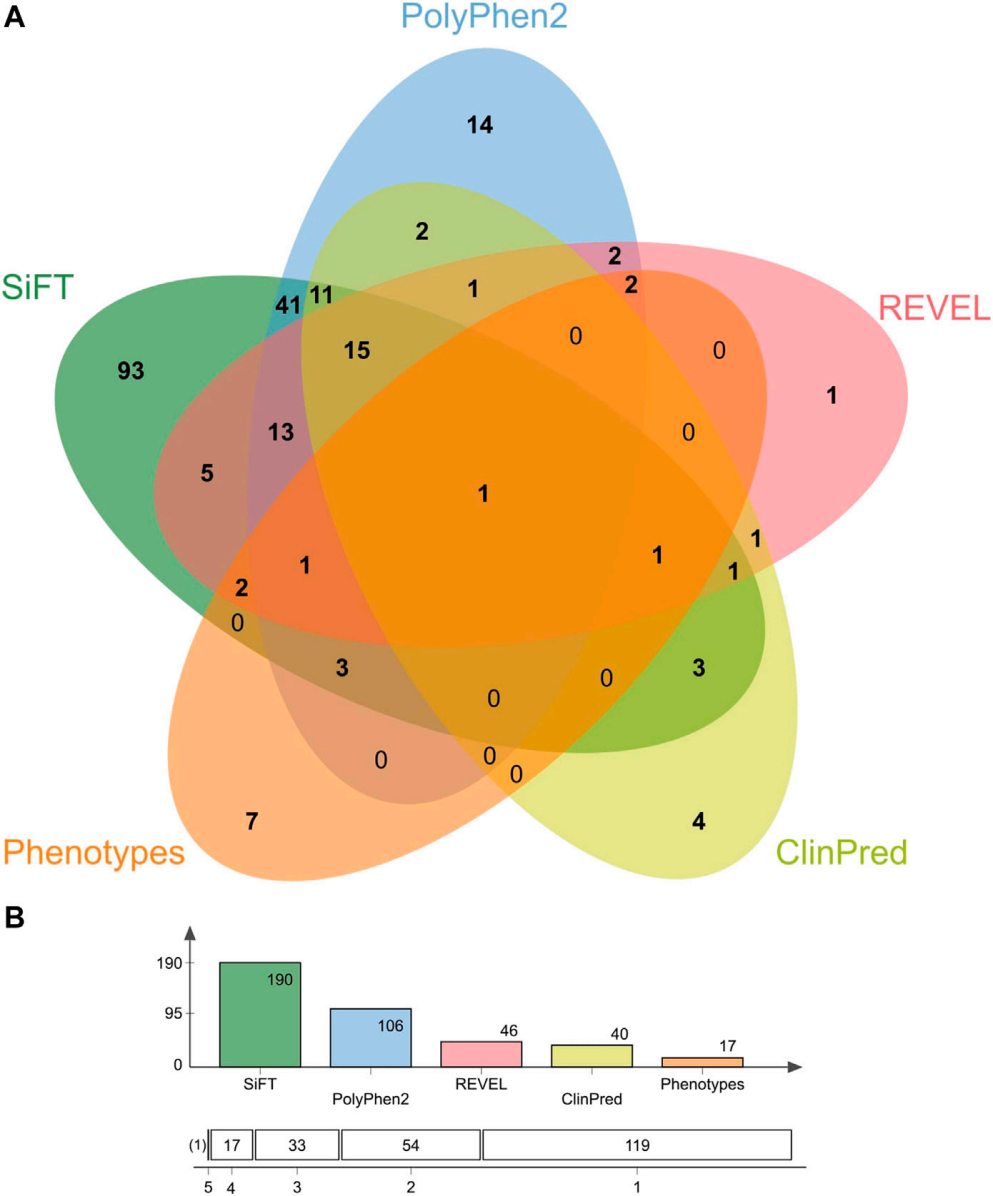
Concordance between sources used to identify variants of interest according to their pathogenicity or association with alterations in the lipid profile. **(A)** Venn diagram with the categorization of missense variants found in genes associated with lipid metabolism and the development of hyperlipidemia. **(B)** The number of variants annotated by shared sources.

Of the missense variants identified within the genes of interest listed in Table 1, 18 were categorized as risk variants by more than three sources used for functional annotation and had more than one count in CR-WGS (Figure 8; Table 5). These variants were located in 16 genes. The alternate allele frequencies ranged from 0.00389 to 0.09143 and 0.00001–0.08852 in CR-WGS and ALL, respectively. Thirteen variants were only present in CR-WGS and ALL; three were reported in AMR and CR-WGS, one in EUR and AMR, one in AFR and AMR, and one in EAS and AMR. In this list, only rs1801689 in *APOH* presented allelic frequencies significantly different from AFR and EAS, and rs202022169 in *CELSR2* showed statistical differences with ALL. Additionally, only nine variants had a phenotype association in ClinVar, GWAS, or Teslovich et al. (2010), including sitosterolemia, cholesterol levels, hypertriglyceridemia, apolipoproteinemia, familial hypercholesterolemia, among others.

**TABLE 5:** Allele frequency and annotation of variants that produce alterations in genes involved in lipid metabolism that are categorized as risky by more than three sources and with more than one count in CR-WGS. CR-WGS: Costa Rican genomes evaluated in this study, ALL: all Subjects from 1KGP phase 3, EAS: East Asia, EUR: Europe, AFR: Africa, AMR: Latin America, S: SIFT, P: PolyPhen2, R: REVEL, C: ClinPred. * Significantly different allelic frequency (*p* < 0.05) compared to CR-WGS.

**Gene**	**dbSNP rsID**	**Alleles (REF/ALT)**	**Allele frequency**						**Annotation**	
			**CR-WGS**	**1KGP Phase 3**						
				**ALL**	**AFR**	**EUR**	**AMR**	**EAS**	**Classified as functional**	**Phenotype association**
ABCA1	rs766619359	C/T	0.00778	-	-	-	-	-	S, P, R, C	
ABCG8	rs11887534	G/C	0.05038	0.06050	0.07639	0.07952	0.09654	0.01388	S, P	**ClinVar:** SITOSTEROLEMIA **GWAS:** C-reactive protein levels or LDL-cholesterol levels (pleiotropy) **Teslovich:** Cholesterol, total | Low-density lipoprotein cholesterol
ABCG8	rs200433692	C/T	0.00581	0.00039	-	-	0.00288	-	S, P, C	
APOA5	rs3135506	G/C	0.09143	0.05571	0.06732	0.06759	0.11671	-	S, P	**ClinVar:** Familial hypertriglyceridemia **GWAS:** Low density lipoprotein cholesterol levels | High density lipoprotein cholesterol levels | Total cholesterol levels | Total triglycerides levels
APOE	rs7412	C/T	0.06614	0.07507	0.10287	0.06262	0.04755	0.10019	S, P, R	**ClinVar:** Apolipoproteinemia E1 | atorvastatin response - Efficacy, Familial type 3 hyperlipoproteinemia | Hypercholesterolemia **GWAS:** Cholesterol, total | HDL cholesterol | High density lipoprotein cholesterol levels | LDL cholesterol | Lipid metabolism phenotypes | Lipoprotein A levels | Lipoprotein-associated phospholipase A2 activity change in response to darapladib treatment in | Response to statins (LDL cholesterol change) | Triglyceride levels
APOH	rs1801689	A/C	0.03112	0.01637	0.00151*	0.04075	0.03602	0.00099*	S, P, R	
APOL1	rs775820342	G/A	0.00389	-	-	-	-	-	S, P, C	
CD36	rs146027667	G/T	0.00389	-	-	-	-	-	S, P, R	
CELSR2	rs202022169	T/C	0.01937	0.00079*	-	-	0.00432	-	S, P, R	
CELSR2	rs1203365203	G/A	0.00387	-	-	-	-	-	S, P, C	
CREB3L3	rs779860332	C/A	0.00389	-	-	-	-	-	S, P, R, C	
GCKR	rs146175795	G/A	0.01162	0.00439	-	-	0.02161	0.00694	S, R, C	**ClinVar:** Hypertriglyceridemia
LCAT	rs4986970	A/T	0.00778	0.00838	0.00151	0.02683	0.00720	-	S, P, R	**ClinVar:** LCAT deficiency **GWAS:** Apolipoprotein A1 levels, Total cholesterol levels
LDLR	rs148698650	G/A	0.00389	0.00079	0.00075	-	0.00288	-	S, R	**ClinVar:** Familial hypercholesterolemia
LIPE	rs1166099993	G/A	0.00389	-	-	-	-	-	S, P, C	
LPL	rs118204057	G/A	0.00583	0.00019	-	-	0.00144	-	P, R	**ClinVar:** Hyperlipidemia, familial combined, LPL related | Hyperlipoproteinemia, type I **GWAS:** High density lipoprotein cholesterol levels | Triglyceride levels
PPARA	rs1800206	C/G	0.03501	0.02276	0.00529*	0.05864	0.03458	-	S,R	**ClinVar:** HYPERAPOBETALIPOPROTEINEMIA, SUSCEPTIBILITY TO
SCARB1	rs748231262	G/A	0.00389	-	-	-	-	-	S, P, R, C	**ClinVar:** Familial hypercholesterolemia

Finally, only eight variants previously linked to lipid metabolism or the development of dyslipidemia in Costa Ricans and Latin Americans were found in CR-WGS (Table 6). These variants were in *ABCA1, ABCG8, CELSR2,* and *LPL* genes, with frequencies ranging from 0.004 to 0.031. The variant rs1231383321 in *LPL* is a private variant found in one individual (heterozygous, sequencing depth 16:21) from CR-WGS.

**TABLE 6:** Variants previously reported in genes involved in lipid metabolism from Costa Rica and Latin America. CR-WGS: Costa Rican genomes evaluated in this study, ALL: all Subjects from 1KGP phase 3, EAS: East Asia, EUR: Europe, AFR: Africa, AMR: Latin America. * Significantly different allelic frequency (*p* < 0.05) compared to CR-WGS. ** Found in one individual.

**Gene**	**dbSNP rsID**	**Alleles (REF/ALT)**	**Frequency of alternative allele**						**Phenotypic association with Latin American populations**
			**CR-WGS**	**1KGP Phase 3**					
				**ALL**	**EUR**	**EAS**	**AFR**	**AMR**	
ABCA1	rs9282541	G/A	0.05252	0.00599*	-	-	0.00075*	0.04178	Allele A found mostly in Native Americans and their descendants. Negative correlation between the early development of coronary disease and HDL-C levels (Villareal-Molina et al. 2012).
ABCG8	rs4245791	C/T	0.74806	0.84105*	0.68986	0.99603*	0.89334*	0.80259	A GWAS shows an association between the C allele with levels of LDL in Latin Americans (Andaleon, Mogil & Wheeler 2019).
CELSR2	rs12740374	G/T	0.21511	0.19548	0.21272	0.04265*	0.24735	0.20461	A GWAS shows an association between the T allele with levels of LDL and cholesterol in Latin Americans (Andaleon, Mogil & Wheeler 2019).
LPL	rs1231383321	C/A	0.00194**	-	-	-	-	-	Allele A found in Costa Ricans with severe hyperlipidemia (González Cordero 2018).
LPL	rs118204057	G/A	0.00583	0.00019	-	-	-	0.00144	Allele A found in Costa Ricans with severe hyperlipidemia (González Cordero 2018).
LPL	rs268	A/G	0.03307	0.00519*	0.01391	-	0.00075*	0.01152	Allele A found in Costa Ricans with severe hyperlipidemia (González Cordero 2018).
LPL	rs316	C/A	0.19455	0.15255	0.12027	0.11210*	0.23676	0.14553	Allele A found in Costa Ricans with severe hyperlipidemia (González Cordero 2018).
LPL	rs328	C/G	0.07198	0.09245	0.13021	0.12202	0.06127	0.06340	Allele G is associated in Costa Ricans with a lower risk for myocardial infarction (Yang et al. 2004).

In summary, we identified 40 variants of interest related to dyslipidemia in CR-WGS. Subjects in our sample presented on average 7.49 of these variants (Figure 5D). Moreover, 60% of the subjects have two or three variants in homozygous state and 20% of the subjects present five variants in heterozygous states.

## 4 Discussion

### 4.1 Exome quality metrics

The bioinformatics workflow used to perform variant calling on the PSYCH-CV and dbGAP-CV cohorts revealed metrics (Ti/Tv and HET/non-ref HOM ratios) within expected values for adequate quality exomes (Wang et al., 2015)​​. Although Ti/Tv ratios were lower than the standard (Wang et al., 2015)​​, we must consider that the exome regions included mature transcripts, miRNAs, and lncRNAs coordinates in Ensembl 106 that could impact lowering the values of this metric. Moreover, HET/non-ref HOM ratios for both cohorts were within the standard for Asians and Africans since this metric is sensitive to ancestry (Wang et al., 2015)​​.

On average, each individual from CR-WGS contained 137k SNVs per exome (210 Mb), but the regions included non-coding sequences that can accumulate more variants. According to the literature, the expected count of SNVs per exome (33 Mb) ranges between 15,000 and 20,000, the determining factor of this variation being the coordinates used to define the exome and the ancestry (Ng et al., 2009; Stitziel et al., 2011). In contrast, there are three million SNPs in a genome (Stitziel et al., 2011). Moreover, the average Ti/Tv ratio, HET/non-ref HOM ratio, and SNV per individual were almost identical in PSYCH-CV and dbGAP-CV (t-test *p*-value >0.05), confirming the possibility of adding both cohorts for variant annotation.

### 4.2 Concordance with the ancestry of Costa Ricans from the Central Valley

The results obtained from the ancestry analysis showed that PSYCH-CV and dbGAP-CV samples show a genetic admixture consistent with Latin American populations and ancestry studies from the Central Valley (Campos-Sánchez et al., 2013). There is also a high concordance between the allele frequencies reported for CR-WGS to the sample of Costa Ricans from the Central Valley without diagnosed disease studied in the Costa Rica Heart Study. All this suggests that the allelic frequencies obtained from CR-WGS are representative of the general population of the Central Valley of Costa Rica and that conclusions from this study can have implications in healthcare policies.

CR-WGS presented an ancestry profile similar to some Latin American groups reported in 1KGP. Of the four Hispanic groups included in 1KGP, the Costa Rican group closely resembles the EUR and EAS component of Colombians (AFR also for PSYCH-CV), and the AFR and EAS component of Mexicans only for PSYCH-CV. This is consistent with previous studies as reviewed by (Adhikari et al., 2017; Wang et al., 2019). The impact of this finding in the study of dyslipidemias in Latin America should be studied further to determine whether conclusions derived from Costa Rican populations apply to other Latin American groups with high European ancestry.

PSYCH-CV and dbGAP-CV samples have comparable admixture proportions to Central Valley samples from Campos-Sánchez et al. (2013), which is consistent with the origin of both cohorts. Notably, the European component was lower in CR-WGS (mean 0.47) and the Asian (used as a proxy of Amerindian) was higher (mean 0.46) compared to Campos-Sánchez et al. (2013) (EUR 0.569 and EAS 0.364). This may be because, in the present study, the East Asian population (EAS) reported in 1KGP was used as the ancestral group instead of an Amerindian group, as in the study by Campos-Sánchez et al. (2013). Although EAS has been used in previous ancestry studies as a group analogous to Native Americans due to their historical origin and because EAS is a broad and standardized group (Wang et al., 2019), it is recommended in future studies to use genomic information from Native Americans for ancestry estimations.

### 4.3 Pharmacogenomic variants

According to the functional annotation extracted from ClinVar and GWAS Catalog, at least nine identified variants have been reported to impact either the efficacy, safety, or metabolism of therapeutic agents (Table 3). Eight of these variants are found in PharmGKB, but three have no conclusive evidence, or no association was found with a pharmacogenomics phenotype.

Four variants in *APOB* showed phenotypes associated with response to warfarin, according to ClinVar; they all presented frequencies above 34%. The same variants are reported in PharmGKB, but only two have a significant association with warfarin. Variants rs1042034 and rs693 were studied in Korean patients under warfarin treatment and the risk of hemorrhage, but the T and G alleles, respectively, were not associated (Yee et al., 2019). However, in the same study, the G allele in rs1367117 and the G allele in rs6789899 were associated with an increased risk of hemorrhage when using warfarin in people with heart valve replacement.

It has been observed in previous studies that the variants rs429358 and rs7412 in *APOE* can alter the efficacy of statin-type drugs such as lovastatin, atorvastatin, or pravastatin to reduce blood cholesterol levels (Mega et al., 2009; Ciuculete et al., 2017; Guan et al., 2019). A study in hypercholesterolemic Chilean patients showed that these variants impact statins response (Lagos et al., 2015). Campos et al. (2001) studied the interaction of *APOE* genotypes (using the *HhaI* enzyme) and fat plasma with lipoprotein levels and low-density lipoproteins in Costa Ricans. Moreover, rs7412 has shown protective effects against SARS-CoV-2 (Espinosa-Salinas et al., 2022). Due to their high allelic frequencies, these variants are candidates for further pharmacogenomic studies in Costa Ricans and Latin American populations (Table 4). On the other hand, rs769450 is an intron variant interpreted as a drug response to warfarin in ClinVar but without assertion criteria. However, in dbSNP, this variant is supported by Musunuru et al. (2012) and Son et al. (2015) associated with decreased risk of elevated triglycerides and LDL (low-density lipoprotein) phenotype, respectively. Additionally, in PharmGKB, allele A is not associated with the risk of hemorrhage during warfarin treatment in people with heart valve replacement compared to allele G.

In *HMGCR*, the genotype TT in rs17238540 is associated with reduced LDL cholesterol in patients treated with simvastatin (Krauss et al., 2008). Furthermore, the genotype GT, compared to TT, showed a decreased reduction in total cholesterol under pravastatin treatment (Chasman et al., 2004). This marker should be studied in more detail in patients under statin treatment.

The only protective variant found was rs3816873 in *MTTP.* This is a microsomal triglyceride transfer protein that catalyzes the transport of triglyceride, cholesteryl ester, and phospholipid between phospholipid surfaces. This variant was associated with protection against metabolic syndrome in ClinVar and OMIM (https://omim.org/entry/157147#0009) and is a benign variant in abetalipoproteinemia.

### 4.4 Risk variants

Alterations in the expression levels or the functioning of the genes involved in lipid metabolism evaluated in this study can cause imbalances in the lipid profile and lead to the development of dyslipidemia. Eight variants presented high impact in VEP; only two were homozygous for the recessive allele (Table 3). For instance, rs132642 in *APOL3* had no annotation in ClinVar, and rs328 in *LPL* is annotated as benign in the phenotype hyperlipoproteinemia type I. This mutation truncates the last two codons of the protein. Evidence from Kobayashi et al. (1992) was from a heterozygous individual and performed expression studies in Cos-1 cells. Faustinella et al. (1991) presented the case of two homozygous brothers in rs328 with another mutation Asp156Gly in LPL. They confirmed *in vitro* that the carboxyl terminus of LPL was not responsible for hyperlipoproteinemia type I. The minor allele frequencies of rs132642 and rs328 are 5.8% and 9.25% in dbSNP (1KGP Global group). All other five high-risk variants identified in Costa Ricans are presented as heterozygous, and only two have ClinVar annotations with uncertain or conflicting interpretations (*CD36, GCKR,* and *GPD1*). In dbSNP, five of these variants (rs5164, rs192225524, rs146053779, rs144009925, and rs749801989) have frequencies below 0.1% in the Global populations of 1KGP and gnomAD exomes. These deserve further study in Latin American populations because of their low allelic frequencies in the same databases (0.3%).

Sixteen out of the 69 genes evaluated contained risk variants defined by more than three bioinformatic tools (Figure 8; Table 4). The genes of the apolipoprotein family with risk variants include *APOA5, APOE, APOH,* and *APOL1*. According to Su & Peng (Su and Peng, 2020), APOA5 and APOE participate in the assembly of VLDLs. The study by Zhou et al. (2018) reported that variants in *APOA* tend to impact plasma triglyceride levels more than cholesterol. Several studies have linked the presence of the C allele in SNV rs3135506 with elevated plasma triglyceride levels (Ruiz-Narváez et al., 2005; Li et al., 2014). Surendran et al. (2012) found an allele frequency of 21% in patients with severe hypertriglyceridemia, while the control group presented a frequency of 9%. This variant reached an allelic frequency of 9% in the Costa Rican group and did not show significant differences with the other 1KGP groups.

On the other hand, several studies have associated the presence of the T allele of the rs7412 variant belonging to *APOE* with high blood cholesterol levels, mainly provided by LDLs, and with high body mass index (Thompson et al., 2009; Tejedor et al., 2014). Although the frequency of this variant in Costa Ricans is 6.6% while that of Latin Americans registered in 1KGP is 4.75%, no statistically significant differences were found between them; evaluating this in other parts of the country or increasing the size of the sample can help clarify whether this trend dissipates or becomes more robust. Although little is known about the molecular role of APOH in lipid metabolism, it has been observed in various populations that the presence of some variants associated with the functioning of this apolipoprotein affects LDL cholesterol levels (Willer et al., 2013). The C allele of the rs1801689 variant has been linked to changes in blood LDL levels; this variation alters the affinity of APOH with phospholipids (Mather et al., 2016). The variant rs775820342 in *APOL1* presented low frequencies in CR-WGS and ALL and is not reported in ClinVar. This is a missense variant with computational pathogenic evidence that could be studied further.

Five risk variants were identified in three genes involved in lipid transport, *ABCA1, ABCG5,* and *ABCG8,* from the ABC transporter family. ABCA1 participates in the formation of HDLs by translocating cholesterol and phospholipids from the interior of the cell to nascent HDLs. The variant rs766619359 in this gene is a missense mutation. The alternate T allele is almost absent in 1KGP (0.004%) and gnomAD (0.0064% genomes, 0.0024% exomes); no reports are available in ClinVar, suggesting that this is a pathogenic variant.

On the other hand, ABCG5 forms a heterodimer with ABCG8 that mediates the absorption and excretion of sterols at multiple levels (Feingold, 2000). Of the risk variants identified, only rs11887534 in *ABCG8* has been associated with changes in the levels of HDLs in the blood in response to statin treatment (Sałacka et al., 2021). Additionally, rs200433692 in *ABCG8* is a missense mutation almost absent in population databases such as 1KGP (0.04%), gnomAD (0.0071% genomes, 0.0088% exomes), and ExAC (0.0116%).

Risk variants were found in four genes (*CELSR2, CREB3L3, GCKR,* and *LCAT*) with a regulatory or signaling role in lipid metabolism. No previous research was found associating the presence of the risk variants found in *CELSR2* and *CREB3L3* with alterations in the lipid profile or risk of suffering from dyslipidemia. Moreover, alternate allele frequencies of the variants rs1203365203 and rs779860332 were extremely low in ALL (0.001%–0.02%) and CR-WGS (0.4%, Table 5). Allele C in rs202022169, on the other hand, presented a statistical difference in the allele frequency with ALL, reaching up to 1.9% in CR-WGS compared to 0.007% in ALL and 0.4% in AMR. However, variant rs146175795 in *GCKR* is presented in ClinVar with conflicting interpretations of pathogenicity, including one associated with hypertriglyceridemia in two heterozygous individuals (Rees et al., 2012). LCAT rs4986970 was reported as benign in ClinVar and it was associated with a reduction in HDL cholesterol (Haase et al., 2012), it presented a frequency of 0.7 in CR-WGS.

Five putative risk variants (0.3–3.5% frequency in CR-WGS) were found in CD36, LDLR, LIPE, PPARA, and SCARB1 genes, involved in lipid and lipoprotein sensing. Variant rs148698650 detected in LDLR has been linked to alterations in lipid profile according to ClinVar, rs1800206 in PPARA has been associated with lipid-altered phenotypes in three studies (Vohl et al., 2000; Tai et al., 2002; Robitaille et al., 2004), and rs748231262 in SCARB1 has one report in an Argentinian study of familial hypercholesterolemia (Corral et al., 2018). The other two variants have frequencies below 0.4% in CR-WGS and are absent from ALL, AFR, EUR, AMR, and EAS.

Finally, *LPL* variant rs118204057 has multiple reports associated with hyperlipidemia and hyperlipoproteinemia pathology and protein function (Monsalve et al., 1990; Hata et al., 1992; Henderson et al., 1992; Mailly et al., 1997; Gilbert et al., 2001; Soto et al., 2015; Ashraf et al., 2017; Caddeo et al., 2018). Moreover, population frequencies are low (ALL 0.019%, 0.14% AMR, 0.58% CR-WGS), and it was detected in one individual with severe hyperlipidemia from Costa Rica (González-Cordero, 2018). This variant deserves further study in Costa Rica and Latin American countries.

### 4.5 Variants previously reported in the Latin American region

We detected in CR-WGS the *ABCA1* variant rs9282541 that was considered a private variant in Native Americans and their descendants (Villarreal-Molina et al., 2012; Du et al., 2020). Its allelic frequency resembles that observed in Latin Americans reported in 1KGP. Villarreal-Molina et al. (2012) reported in Mexican subjects that this variant was associated with lower levels of total cholesterol and HDL cholesterol in plasma. Additionally, they observed that the variant’s effect depends on the sex of the subject, probably interacting with other factors.

Two variants reported in the study by Andaleon et al. (2019), which focused on identifying variants associated with changes in the lipid profile of Latin Americans living in the United States, were found in the Costa Rican cohort analyzed. The intron variant rs4245791 in *ABCG8* is not annotated in ClinVar. However, several publications provide evidence of its relationship with total cholesterol (Ma et al., 2010); higher cholestanol-to-cholesterol levels -an estimate of cholesterol absorption- (Silbernagel et al., 2013), and increased plasma phytosterol concentrations, relatively elevated LDL-C; and increased coronary artery disease risk (Calandra et al., 2011). According to research, the variant rs12740374 in *CELSR2* influences LDL cholesterol levels in Hispanics (Samani et al., 2007; Consortium et al., 2009; Musunuru et al., 2010).

Although the research by Andaleon et al. (2019) detected genetic variants with a quantitative impact on plasma lipid levels for Latin Americans, it is essential to mention that the people included in that study reside in the United States. This means they were exposed to different lifestyles and environmental conditions than their country of origin. Only the environment can affect the variation of plasma total cholesterol levels up to 21% and 29% in plasma triglyceride levels; approximately 6% of the variation is attributed to the interaction between environment and genetics (Elder et al., 2009).

We detected in CR-WGS four of the 15 variants described by González Cordero (2018) in *LPL* (Table 6). According to a meta-analysis, the G allele in the rs268 variant is associated with lower plasma HDL cholesterol levels (Boes et al., 2009). This variant has a frequency of 3.3% in CR-WGS, significantly higher compared to ALL and AFR but not to AMR (1.1%) and EUR (1.3%). Variant rs316 is intronic, and according to Pirim et al. (2014), it is possibly located next to a regulatory site. The A allele in this variant has been repeatedly associated with an increase in HDL cholesterol (Schuster et al., 2011; Pirim et al., 2014, 2015), but it is benign in ClinVar. The missense variant rs1231383321 was detected in one individual in CR-WGS, and it is also reported in American gnomAD-exomes and genomes with a frequency of 0.023% and 0.051%, respectively. The rs118204057 variant was discussed previously.

On the other hand, we identified the *LPL* variant rs328 (S447*) in CR-WGS, this was previously associated in a publication of the Costa Rica Heart Study with a reduction in the risk of myocardial infarction in Costa Ricans (Yang et al., 2004). The G allele suppresses the encoding of the last two amino acids of LPL, increasing its lipase activity. Notably, this is associated with low levels of plasma triglycerides and increases in HDL cholesterol in healthy subjects. However, in subjects with obesity, this allele instead is associated with elevated levels of plasma triglycerides (Palacio-Rojas et al., 2017).

Overall, this study presents the reanalysis of Costa Ricans’ genomic data to estimate dyslipidemia variants’ baseline frequencies. The finding that these genomes’ ancestry accurately resembles those of Central Valley and some Latin American populations is relevant, considering the low amount of genomic data in these populations to derive conclusions about the genetic burden in the general population.

The study identified 2,600 variants in 69 genes involved in lipid metabolism in the genomes of people from the Central Valley of Costa Rica. Among these, 33 variants have the potential to affect the functioning of these genes, some have been directly linked to the development of hyperlipidemia, and some could affect the performance of proteins involved in lipid metabolism according to bioinformatic analysis. However, some have not been directly associated with developing such conditions in the literature. On the other hand, we found seven variants with pharmacogenomic relevance, several of which can modulate the subject’s response to the application of statin-type drugs, therapies commonly used to treat cases of severe hyperlipidemia. Our analysis of the number of variants per individual for the 40 variants of interest suggests an important genetic burden for dyslipidemia in our sample; however, we could not determine the relationship of these variants with dyslipidemia phenotypes due to the lack of metadata associated with the datasets analyzed.

In the future, it is essential to develop studies that capture environmental, genotypic, and phenotypic data from Costa Ricans living in Costa Rica to understand more clearly the dynamics that participate in the incidence of dyslipidemia. These efforts can be focused on the 23 genes and 40 variants identified in this study, which can be analyzed with traditional genotyping methodologies (i.e., PCR, RFLP, Sanger sequencing) reducing costs. Alternatively, genetic analysis using genome sequencing, exome sequencing, or a panel of genes involved in lipid metabolism, such as the LipidSeq panel described by Johansen et al. (2014), could help to identify variants in affected individuals. In an Argentinian study, this strategy has already been used (Corral et al., 2018), where they sequenced only genes linked to lipid metabolism. Additionally, copy number variants should be studied as they have been involved in certain dyslipidemia disorders (Iacocca and Hegele, 2018). Moreover, the abundant clinical information hosted in the Costa Rican Social Security System (Caja Costarricense del Seguro Social - C.C.S.S.) could strengthen this type of genomic study. Eventually, functional validation of the variants detected in patients should be performed to provide conclusive evidence of the association with dyslipidemia.

## Data Availability

Publicly available datasets were analyzed in this study. This data can be found here: phs000988.V4.P1 can be requested directly through dbGAP. Chavarria-Soley et al. 2021 can be requested through the original authors.
