# Supplementary material for: Frequencies of variants in genes associated with dyslipidemias identified in Costa Rican genomes
Source: Front Genet. 2023 Mar 30;14:1114774. doi: 10.3389/fgene.2023.1114774 (PMC10098023; doi:10.3389/fgene.2023.1114774)
Supplement: Supplementary file 1 [file DataSheet1.docx]

***Supplementary Material***

**Supplementary Figure 1.** Exome quality metrics by chromosome in PSYCH-CV and dbGAP-CV. (A) number of SNPs identified per chromosome individual, (B) number of indels identified per chromosome individual, (C) T_I_/T_V_ ratio per chromosome per individual calculated from variants reported in dbSNP, (D) HET/non-ref HOM ratio per chromosome per individual.

**
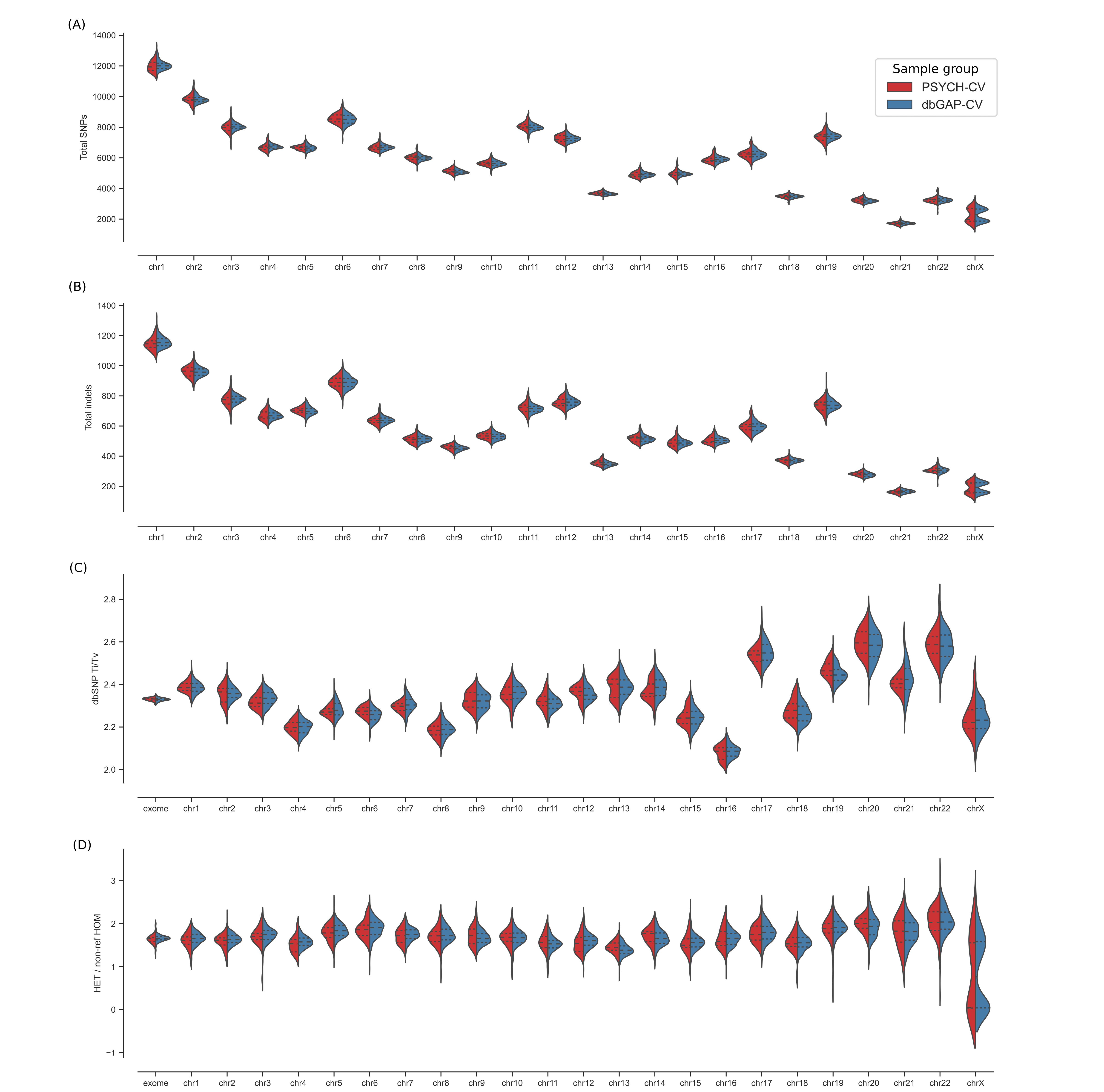
**

**Supplementary Figure 2.** Linear relationship between allelic frequencies of 15 SNPs obtained in CR-WGS cohort and healthy Costa Ricans from the Central Valley from the Costa Rica Heart Study (Brown et al., 2003; Ruiz-Narvaez et al., 2005, 2010).


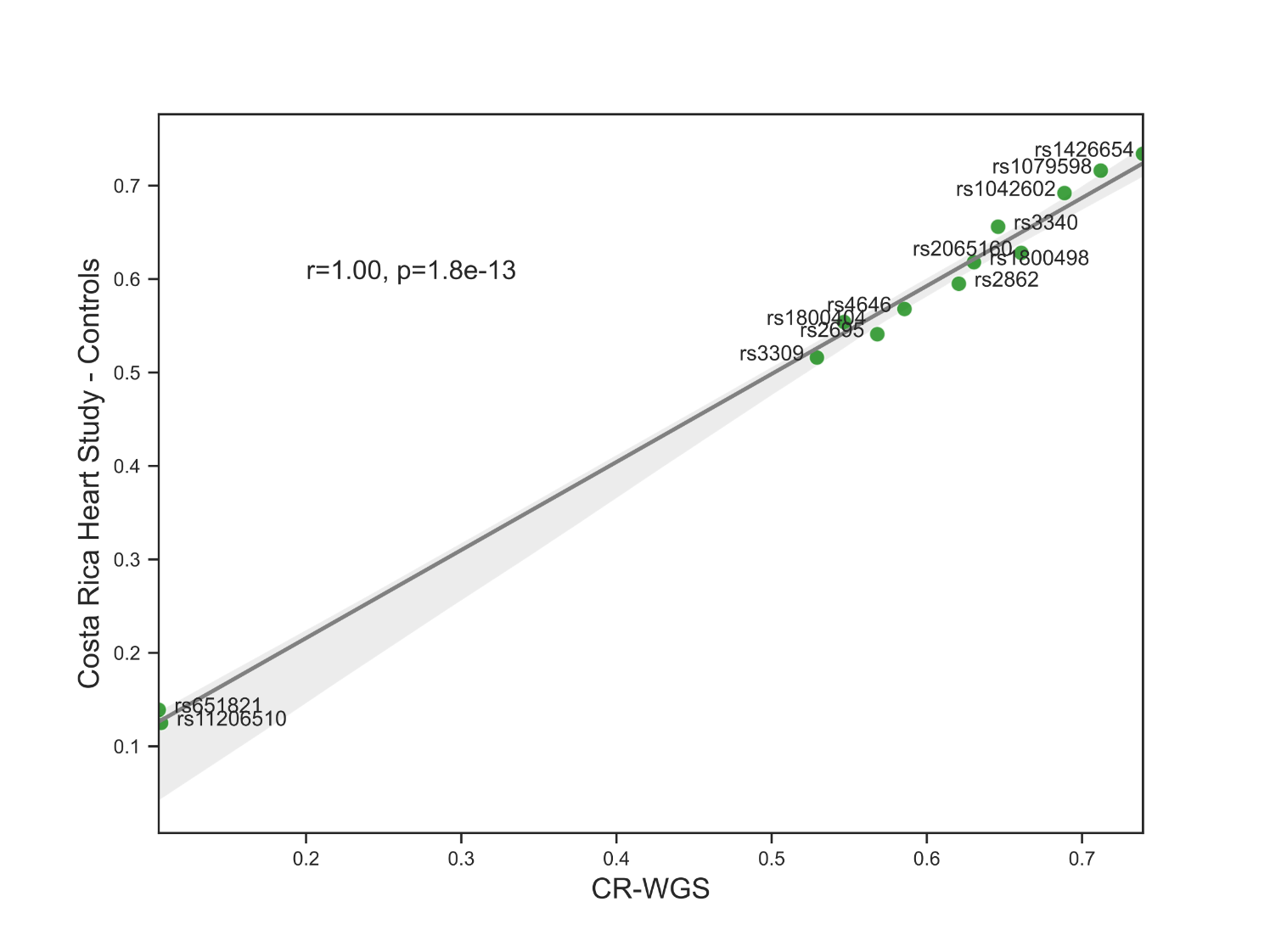


**Supplementary Figure 3.** Counts of variants detected in genes involved in lipid metabolism, categorized by their impact on the transcript according to Variant Effect Predictor (VEP).


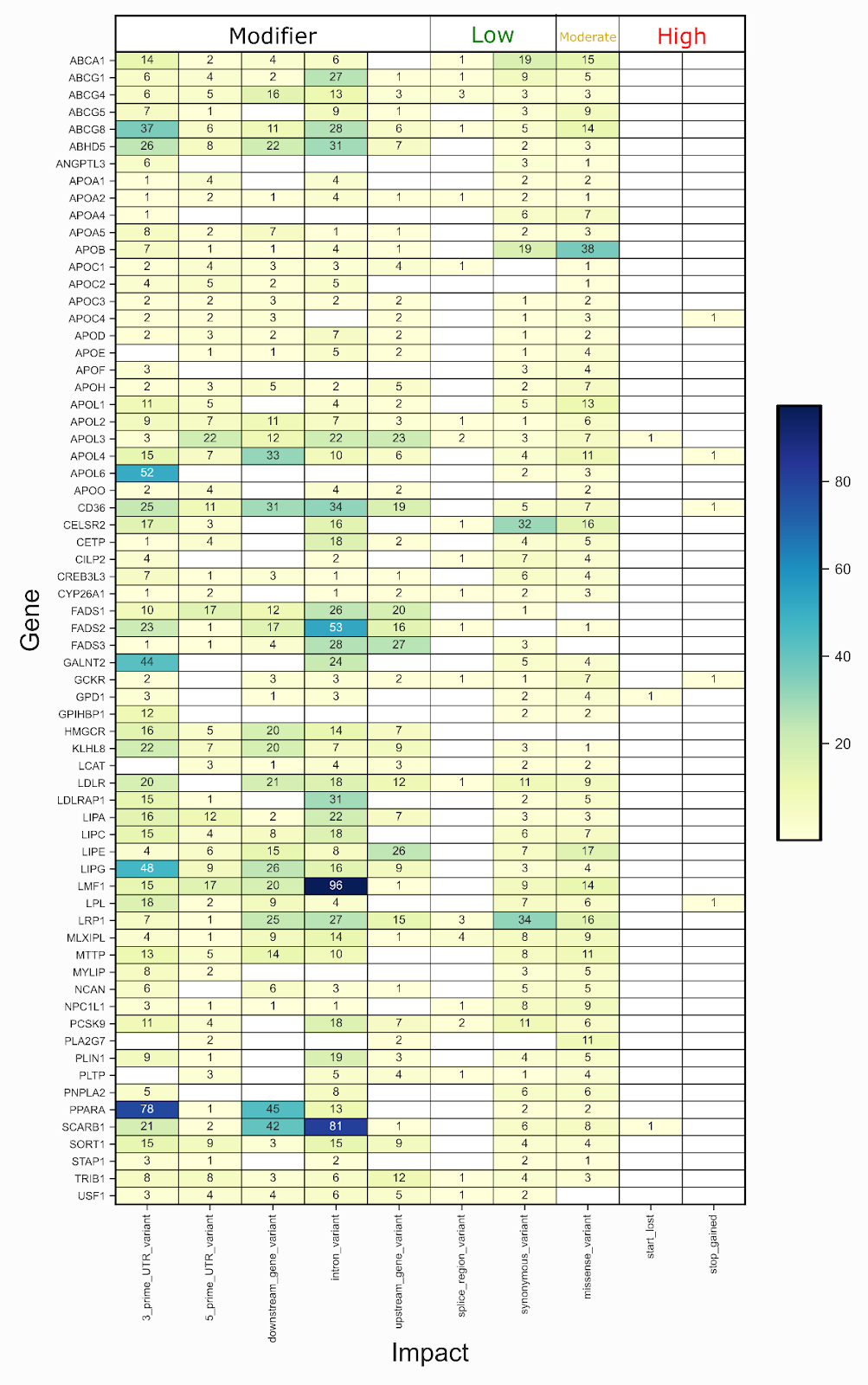


**Supplementary Figure 4.** Differences observed between allelic frequencies in genes associated with lipid metabolism in Costa Ricans compared to those reported in 1KGP. (A) Probability according to Fisher's tests that the polymorphic sites in CR-WGS have differences in their allele frequencies with respect to (A) AFR, (B) EAS. The dotted line marks the significance threshold with the Bonferroni fit. Variants are categorized as LOW, MODERATE and HIGH by VEP. CR-WGS: Costa Rican genomes evaluated in this study, ALL: all Subjects from 1KGP phase 3, EAS: East Asia, EUR: Europe, AFR: Africa, AMR: Latin America.


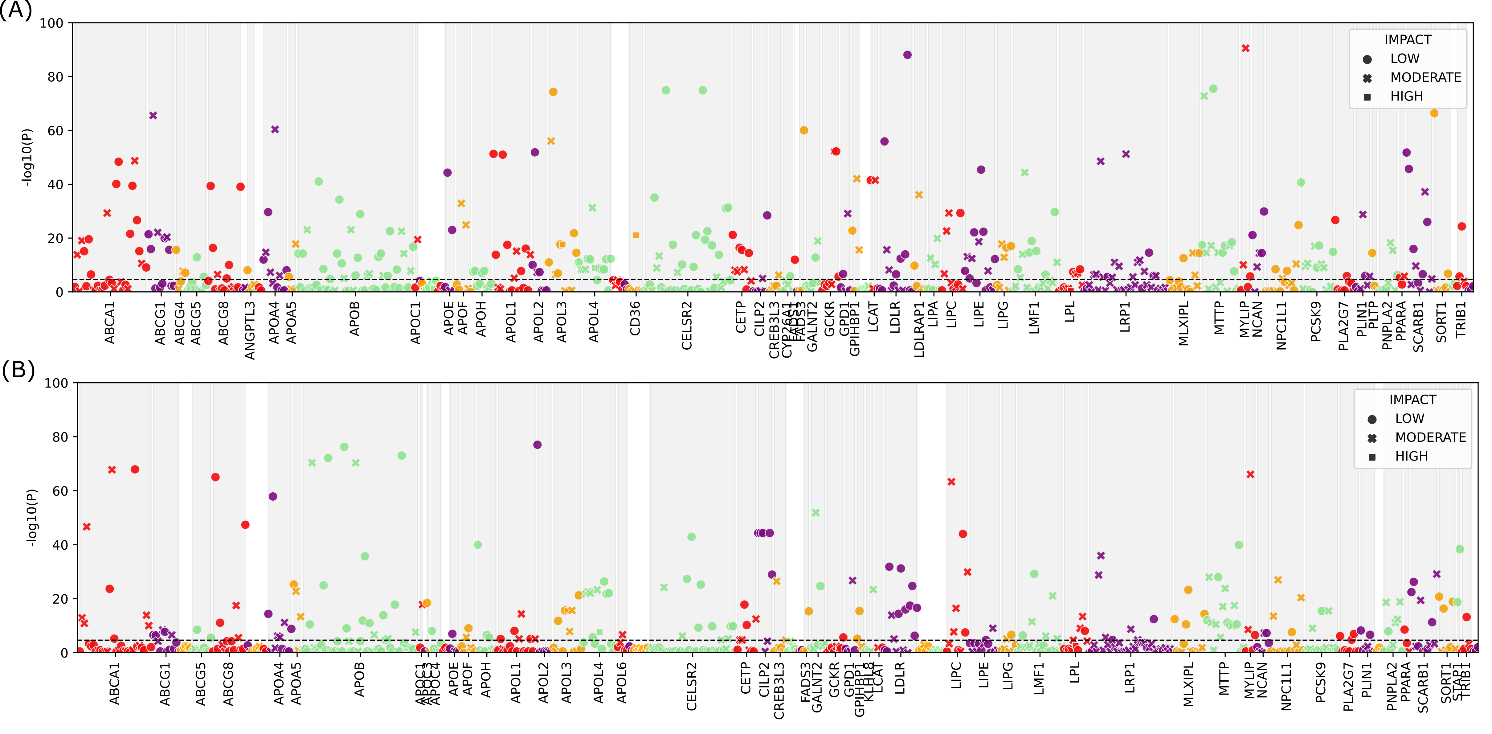


**Supplementary Table 1.** Samples selected for analysis from the project dbGAP phs000988.V4.P1, these are called dbGAP-CV.

| **No.** | **Sample** | **No.** | **Sample** | **No.** | **Sample** | **No.** | **Sample** | **No.** | **Sample** | **No.** | **Sample** |
| --- | --- | --- | --- | --- | --- | --- | --- | --- | --- | --- | --- |
| 1 | NWD101850 | 46 | NWD268247 | 91 | NWD401180 | 136 | NWD576325 | 181 | NWD767610 | 226 | NWD969419 |
| 2 | NWD120416 | 47 | NWD268495 | 92 | NWD401962 | 137 | NWD592749 | 182 | NWD770924 | 227 | NWD971027 |
| 3 | NWD121699 | 48 | NWD268891 | 93 | NWD407487 | 138 | NWD593180 | 183 | NWD771767 | 228 | NWD973867 |
| 4 | NWD122673 | 49 | NWD270876 | 94 | NWD411668 | 139 | NWD599946 | 184 | NWD786967 | 229 | NWD978003 |
| 5 | NWD124490 | 50 | NWD271159 | 95 | NWD415466 | 140 | NWD607594 | 185 | NWD788119 | 230 | NWD981775 |
| 6 | NWD130678 | 51 | NWD274196 | 96 | NWD420735 | 141 | NWD609869 | 186 | NWD797364 | 231 | NWD990575 |
| 7 | NWD135836 | 52 | NWD277131 | 97 | NWD421513 | 142 | NWD614013 | 187 | NWD797498 | 232 | NWD994356 |
| 8 | NWD136601 | 53 | NWD280100 | 98 | NWD423586 | 143 | NWD621181 | 188 | NWD805292 | 233 | NWD994436 |
| 9 | NWD138043 | 54 | NWD282137 | 99 | NWD426865 | 144 | NWD627618 | 189 | NWD807987 | 234 | NWD996801 |
| 10 | NWD139385 | 55 | NWD293369 | 100 | NWD428764 | 145 | NWD630361 | 190 | NWD809272 |  |  |
| 11 | NWD152923 | 56 | NWD300786 | 101 | NWD429349 | 146 | NWD633520 | 191 | NWD811284 |  |  |
| 12 | NWD153158 | 57 | NWD301935 | 102 | NWD430931 | 147 | NWD633757 | 192 | NWD822216 |  |  |
| 13 | NWD156380 | 58 | NWD303793 | 103 | NWD435635 | 148 | NWD635742 | 193 | NWD826645 |  |  |
| 14 | NWD161685 | 59 | NWD304983 | 104 | NWD437315 | 149 | NWD639450 | 194 | NWD834686 |  |  |
| 15 | NWD162082 | 60 | NWD305462 | 105 | NWD439370 | 150 | NWD644576 | 195 | NWD842737 |  |  |
| 16 | NWD163082 | 61 | NWD305520 | 106 | NWD443983 | 151 | NWD647073 | 196 | NWD849563 |  |  |
| 17 | NWD164443 | 62 | NWD307070 | 107 | NWD445187 | 152 | NWD652119 | 197 | NWD849612 |  |  |
| 18 | NWD178068 | 63 | NWD307934 | 108 | NWD449443 | 153 | NWD655155 | 198 | NWD851056 |  |  |
| 19 | NWD183430 | 64 | NWD308271 | 109 | NWD451591 | 154 | NWD657109 | 199 | NWD861737 |  |  |
| 20 | NWD183517 | 65 | NWD309126 | 110 | NWD455939 | 155 | NWD658520 | 200 | NWD861942 |  |  |
| 21 | NWD188114 | 66 | NWD312162 | 111 | NWD469263 | 156 | NWD662457 | 201 | NWD867285 |  |  |
| 22 | NWD193683 | 67 | NWD315264 | 112 | NWD478360 | 157 | NWD668871 | 202 | NWD869246 |  |  |
| 23 | NWD194738 | 68 | NWD328903 | 113 | NWD480155 | 158 | NWD673356 | 203 | NWD873855 |  |  |
| 24 | NWD199759 | 69 | NWD330155 | 114 | NWD489083 | 159 | NWD678458 | 204 | NWD874486 |  |  |
| 25 | NWD204671 | 70 | NWD331114 | 115 | NWD491083 | 160 | NWD678602 | 205 | NWD880377 |  |  |
| 26 | NWD204919 | 71 | NWD340293 | 116 | NWD497224 | 161 | NWD678712 | 206 | NWD882738 |  |  |
| 27 | NWD207172 | 72 | NWD343999 | 117 | NWD500480 | 162 | NWD683837 | 207 | NWD889292 |  |  |
| 28 | NWD208102 | 73 | NWD346393 | 118 | NWD502650 | 163 | NWD684446 | 208 | NWD889765 |  |  |
| 29 | NWD209269 | 74 | NWD346468 | 119 | NWD503184 | 164 | NWD687697 | 209 | NWD893155 |  |  |
| 30 | NWD211734 | 75 | NWD347779 | 120 | NWD511161 | 165 | NWD688209 | 210 | NWD894617 |  |  |
| 31 | NWD211815 | 76 | NWD349716 | 121 | NWD519898 | 166 | NWD694068 | 211 | NWD895913 |  |  |
| 32 | NWD219097 | 77 | NWD352933 | 122 | NWD525158 | 167 | NWD695785 | 212 | NWD897943 |  |  |
| 33 | NWD222448 | 78 | NWD353514 | 123 | NWD528909 | 168 | NWD695880 | 213 | NWD902021 |  |  |
| 34 | NWD223189 | 79 | NWD361784 | 124 | NWD529614 | 169 | NWD697885 | 214 | NWD905248 |  |  |
| 35 | NWD229997 | 80 | NWD365731 | 125 | NWD532780 | 170 | NWD705178 | 215 | NWD913101 |  |  |
| 36 | NWD230311 | 81 | NWD366029 | 126 | NWD533352 | 171 | NWD707533 | 216 | NWD918403 |  |  |
| 37 | NWD230375 | 82 | NWD373460 | 127 | NWD533424 | 172 | NWD714294 | 217 | NWD929473 |  |  |
| 38 | NWD241826 | 83 | NWD375551 | 128 | NWD542232 | 173 | NWD723093 | 218 | NWD933744 |  |  |
| 39 | NWD248515 | 84 | NWD376234 | 129 | NWD549645 | 174 | NWD725761 | 219 | NWD940790 |  |  |
| 40 | NWD248657 | 85 | NWD381620 | 130 | NWD553845 | 175 | NWD731524 | 220 | NWD942200 |  |  |
| 41 | NWD250816 | 86 | NWD383240 | 131 | NWD556152 | 176 | NWD734527 | 221 | NWD943005 |  |  |
| 42 | NWD252604 | 87 | NWD383901 | 132 | NWD568742 | 177 | NWD741589 | 222 | NWD945441 |  |  |
| 43 | NWD258909 | 88 | NWD386997 | 133 | NWD572136 | 178 | NWD742087 | 223 | NWD946043 |  |  |
| 44 | NWD259403 | 89 | NWD388907 | 134 | NWD572300 | 179 | NWD742772 | 224 | NWD955901 |  |  |
| 45 | NWD260742 | 90 | NWD396722 | 135 | NWD575181 | 180 | NWD764752 | 225 | NWD966349 |  |  |

**Supplementary Table 2.** Exome quality metrics for each sample in PSYCH-CV and dbGAP-CV

| **Sample name** | **Sample group** | **Ratio HET/non-ref HOM** | **Total variants** | **Total SNPs** | **SNPs in dbSNP** | **Novel SNPs** | **Ti/Tv ratio** | **Total indels** | **Total multiallelic SNPs** |
| --- | --- | --- | --- | --- | --- | --- | --- | --- | --- |
| 8007540210 | PSYCH-CV | 1.56939 | 148973 | 135788 | 135705 | 83 | 2.335013 | 13185 | 1939 |
| 8007540307 | PSYCH-CV | 1.763712 | 155481 | 141900 | 141729 | 171 | 2.33692 | 13581 | 1982 |
| 8007540612 | PSYCH-CV | 1.699364 | 151738 | 138383 | 138283 | 100 | 2.320838 | 13355 | 1935 |
| 8007540751 | PSYCH-CV | 1.63001 | 148439 | 135426 | 135293 | 133 | 2.323091 | 13013 | 1945 |
| 8007540763 | PSYCH-CV | 1.657441 | 152212 | 138852 | 138757 | 95 | 2.328863 | 13360 | 1964 |
| 8007540916 | PSYCH-CV | 1.686639 | 149903 | 136746 | 136651 | 95 | 2.332951 | 13157 | 1923 |
| 8007540984 | PSYCH-CV | 1.673718 | 150219 | 137054 | 136971 | 83 | 2.333114 | 13165 | 1915 |
| 8007540997 | PSYCH-CV | 1.479221 | 147658 | 134593 | 134530 | 63 | 2.339456 | 13065 | 1926 |
| 8007540998 | PSYCH-CV | 1.631832 | 149676 | 136510 | 136437 | 73 | 2.335705 | 13166 | 1932 |
| 8007541322 | PSYCH-CV | 1.59405 | 149866 | 136717 | 136553 | 164 | 2.328369 | 13149 | 1910 |
| 8007541345 | PSYCH-CV | 1.682465 | 151218 | 137893 | 137819 | 74 | 2.335326 | 13325 | 1908 |
| 8007541366 | PSYCH-CV | 1.614909 | 151050 | 137626 | 137499 | 127 | 2.342547 | 13424 | 1951 |
| 8007541415 | PSYCH-CV | 1.652125 | 150529 | 137262 | 137142 | 120 | 2.336545 | 13267 | 1925 |
| 8007541488 | PSYCH-CV | 1.556312 | 150206 | 137012 | 136949 | 63 | 2.308027 | 13194 | 1915 |
| 8007541512 | PSYCH-CV | 1.65757 | 149818 | 136655 | 136587 | 68 | 2.330578 | 13163 | 1918 |
| 8007541524 | PSYCH-CV | 1.31438 | 144381 | 131711 | 131633 | 78 | 2.337551 | 12670 | 1894 |
| 8007541548 | PSYCH-CV | 1.961458 | 163742 | 149216 | 149040 | 176 | 2.329312 | 14526 | 2090 |
| 8007541751 | PSYCH-CV | 1.617855 | 150283 | 136976 | 136862 | 114 | 2.322861 | 13307 | 1961 |
| 8007542276 | PSYCH-CV | 1.5588 | 149176 | 136019 | 135933 | 86 | 2.33308 | 13157 | 1918 |
| 8007542377 | PSYCH-CV | 1.685 | 151611 | 138288 | 138162 | 126 | 2.320563 | 13323 | 1925 |
| 8007542399 | PSYCH-CV | 1.678827 | 150157 | 136931 | 136847 | 84 | 2.325969 | 13226 | 1962 |
| 8007542420 | PSYCH-CV | 1.544135 | 149537 | 136443 | 136336 | 107 | 2.331769 | 13094 | 1907 |
| 8007542432 | PSYCH-CV | 1.741753 | 153132 | 139703 | 139638 | 65 | 2.325348 | 13429 | 2020 |
| NWD101850 | dbGAP-CV | 1.649619 | 149497 | 136232 | 136173 | 59 | 2.338719 | 13265 | 1964 |
| NWD120416 | dbGAP-CV | 1.579688 | 148556 | 135460 | 135417 | 43 | 2.323851 | 13096 | 1917 |
| NWD121699 | dbGAP-CV | 1.676458 | 152917 | 139451 | 139450 | 1 | 2.332616 | 13466 | 1970 |
| NWD122673 | dbGAP-CV | 1.642107 | 148494 | 135499 | 135473 | 26 | 2.317165 | 12995 | 1888 |
| NWD124490 | dbGAP-CV | 1.565831 | 149214 | 136083 | 135958 | 125 | 2.326759 | 13131 | 1929 |
| NWD130678 | dbGAP-CV | 1.617045 | 150966 | 137643 | 137579 | 64 | 2.33226 | 13323 | 1895 |
| NWD135836 | dbGAP-CV | 1.614811 | 150315 | 137029 | 136962 | 67 | 2.331031 | 13286 | 1998 |
| NWD136601 | dbGAP-CV | 1.712507 | 149404 | 136225 | 136164 | 61 | 2.317351 | 13179 | 1923 |
| NWD138043 | dbGAP-CV | 1.708401 | 151059 | 137790 | 137709 | 81 | 2.333793 | 13269 | 1975 |
| NWD139385 | dbGAP-CV | 1.710683 | 150750 | 137636 | 137621 | 15 | 2.338209 | 13114 | 1960 |
| NWD152923 | dbGAP-CV | 1.525312 | 149099 | 135975 | 135831 | 144 | 2.329436 | 13124 | 1995 |
| NWD153158 | dbGAP-CV | 1.647858 | 149646 | 136478 | 136448 | 30 | 2.331331 | 13168 | 2007 |
| NWD156380 | dbGAP-CV | 1.634536 | 148821 | 135663 | 135600 | 63 | 2.329241 | 13158 | 1931 |
| NWD161685 | dbGAP-CV | 1.681039 | 151556 | 138142 | 138141 | 1 | 2.322135 | 13414 | 1947 |
| NWD162082 | dbGAP-CV | 1.674859 | 150747 | 137487 | 137421 | 66 | 2.328271 | 13260 | 1931 |
| NWD163082 | dbGAP-CV | 1.638617 | 150302 | 137196 | 137174 | 22 | 2.314903 | 13106 | 1889 |
| NWD164443 | dbGAP-CV | 1.639892 | 149583 | 136443 | 136439 | 4 | 2.314522 | 13140 | 1921 |
| NWD178068 | dbGAP-CV | 1.682662 | 150335 | 136974 | 136964 | 10 | 2.328975 | 13361 | 1868 |
| NWD183430 | dbGAP-CV | 1.586481 | 150299 | 137114 | 137055 | 59 | 2.324641 | 13185 | 1986 |
| NWD183517 | dbGAP-CV | 1.876958 | 156920 | 143076 | 143072 | 4 | 2.323237 | 13844 | 2060 |
| NWD188114 | dbGAP-CV | 1.668439 | 149386 | 136036 | 136035 | 1 | 2.325958 | 13350 | 1949 |
| NWD193683 | dbGAP-CV | 1.620866 | 149858 | 136564 | 136488 | 76 | 2.335891 | 13294 | 1931 |
| NWD194738 | dbGAP-CV | 1.583586 | 148595 | 135701 | 135682 | 19 | 2.335841 | 12894 | 1961 |
| NWD199759 | dbGAP-CV | 1.76924 | 152934 | 139425 | 139284 | 141 | 2.330241 | 13509 | 2003 |
| NWD204671 | dbGAP-CV | 1.668628 | 148937 | 135721 | 135695 | 26 | 2.340185 | 13216 | 1958 |
| NWD204919 | dbGAP-CV | 1.66836 | 152207 | 138756 | 138756 | 0 | 2.326365 | 13451 | 1970 |
| NWD207172 | dbGAP-CV | 1.689799 | 152625 | 139110 | 139110 | 0 | 2.323855 | 13515 | 1957 |
| NWD208102 | dbGAP-CV | 1.631509 | 148120 | 134965 | 134964 | 1 | 2.334668 | 13155 | 1957 |
| NWD209269 | dbGAP-CV | 1.576878 | 149160 | 136116 | 136089 | 27 | 2.327848 | 13044 | 1921 |
| NWD211734 | dbGAP-CV | 1.695706 | 150313 | 136969 | 136930 | 39 | 2.333658 | 13344 | 1935 |
| NWD211815 | dbGAP-CV | 1.639366 | 149233 | 136107 | 136046 | 61 | 2.324032 | 13126 | 1955 |
| NWD219097 | dbGAP-CV | 1.693356 | 152677 | 139254 | 139160 | 94 | 2.326322 | 13423 | 1993 |
| NWD222448 | dbGAP-CV | 1.635537 | 150156 | 136969 | 136968 | 1 | 2.328182 | 13187 | 1924 |
| NWD223189 | dbGAP-CV | 1.619337 | 149839 | 136566 | 136563 | 3 | 2.335768 | 13273 | 1950 |
| NWD229997 | dbGAP-CV | 1.637663 | 149623 | 136493 | 136492 | 1 | 2.327612 | 13130 | 1914 |
| NWD230311 | dbGAP-CV | 1.692748 | 152294 | 139159 | 139139 | 20 | 2.337147 | 13135 | 1951 |
| NWD230375 | dbGAP-CV | 1.660414 | 151195 | 138002 | 137993 | 9 | 2.327538 | 13193 | 1947 |
| NWD241826 | dbGAP-CV | 1.641471 | 150118 | 136938 | 136869 | 69 | 2.34504 | 13180 | 1909 |
| NWD248515 | dbGAP-CV | 1.686243 | 149120 | 136044 | 135976 | 68 | 2.329073 | 13076 | 1956 |
| NWD248657 | dbGAP-CV | 1.73449 | 151299 | 138044 | 137990 | 54 | 2.331724 | 13255 | 1963 |
| NWD250816 | dbGAP-CV | 1.556199 | 149229 | 136091 | 136090 | 1 | 2.322834 | 13138 | 1917 |
| NWD252604 | dbGAP-CV | 1.638858 | 150393 | 137120 | 137083 | 37 | 2.326531 | 13273 | 1923 |
| NWD258909 | dbGAP-CV | 1.693283 | 152387 | 138930 | 138846 | 84 | 2.320721 | 13457 | 1921 |
| NWD259403 | dbGAP-CV | 1.591919 | 148620 | 135435 | 135434 | 1 | 2.332612 | 13185 | 1966 |
| NWD260742 | dbGAP-CV | 1.596768 | 149205 | 136096 | 136052 | 44 | 2.336407 | 13109 | 1919 |
| NWD268247 | dbGAP-CV | 1.720196 | 150273 | 137136 | 137130 | 6 | 2.335117 | 13137 | 1944 |
| NWD268495 | dbGAP-CV | 1.647244 | 150307 | 137170 | 137116 | 54 | 2.331373 | 13137 | 1934 |
| NWD268891 | dbGAP-CV | 1.705404 | 156089 | 142270 | 142224 | 46 | 2.332256 | 13819 | 2017 |
| NWD270876 | dbGAP-CV | 1.756542 | 153649 | 140132 | 140128 | 4 | 2.326165 | 13517 | 1971 |
| NWD271159 | dbGAP-CV | 1.714399 | 150500 | 137361 | 137344 | 17 | 2.31093 | 13139 | 1935 |
| NWD274196 | dbGAP-CV | 1.56543 | 147716 | 134718 | 134713 | 5 | 2.325754 | 12998 | 1906 |
| NWD277131 | dbGAP-CV | 1.583712 | 147287 | 134443 | 134442 | 1 | 2.337272 | 12844 | 1866 |
| NWD280100 | dbGAP-CV | 1.645952 | 151432 | 138073 | 137981 | 92 | 2.315018 | 13359 | 1965 |
| NWD282137 | dbGAP-CV | 1.639111 | 150271 | 137066 | 137065 | 1 | 2.326901 | 13205 | 1937 |
| NWD293369 | dbGAP-CV | 1.664525 | 151851 | 138442 | 138156 | 286 | 2.331388 | 13409 | 1951 |
| NWD300786 | dbGAP-CV | 1.690119 | 149900 | 136657 | 136613 | 44 | 2.330643 | 13243 | 1890 |
| NWD301935 | dbGAP-CV | 1.676921 | 154511 | 140828 | 140658 | 170 | 2.320225 | 13683 | 1974 |
| NWD303793 | dbGAP-CV | 1.693387 | 150381 | 137177 | 137081 | 96 | 2.328178 | 13204 | 1946 |
| NWD304983 | dbGAP-CV | 1.587232 | 148584 | 135568 | 135532 | 36 | 2.332809 | 13016 | 1927 |
| NWD305462 | dbGAP-CV | 1.624573 | 148938 | 135724 | 135679 | 45 | 2.32286 | 13214 | 1962 |
| NWD305520 | dbGAP-CV | 1.690557 | 151469 | 137927 | 137885 | 42 | 2.324533 | 13542 | 1956 |
| NWD307070 | dbGAP-CV | 1.578177 | 149342 | 136229 | 136220 | 9 | 2.319524 | 13113 | 1925 |
| NWD307934 | dbGAP-CV | 1.691389 | 151312 | 138042 | 138034 | 8 | 2.329329 | 13270 | 1963 |
| NWD308271 | dbGAP-CV | 1.642815 | 148990 | 135856 | 135822 | 34 | 2.348834 | 13134 | 1966 |
| NWD309126 | dbGAP-CV | 1.659813 | 151748 | 138382 | 138299 | 83 | 2.317875 | 13366 | 1983 |
| NWD312162 | dbGAP-CV | 1.766107 | 157924 | 144003 | 144001 | 2 | 2.320673 | 13921 | 2020 |
| NWD315264 | dbGAP-CV | 1.620907 | 149606 | 136598 | 136591 | 7 | 2.345359 | 13008 | 1927 |
| NWD328903 | dbGAP-CV | 1.681001 | 152337 | 138774 | 138689 | 85 | 2.335233 | 13563 | 1999 |
| NWD330155 | dbGAP-CV | 1.6888 | 151214 | 137923 | 137836 | 87 | 2.311057 | 13291 | 1989 |
| NWD331114 | dbGAP-CV | 1.703835 | 149304 | 136212 | 136200 | 12 | 2.331866 | 13092 | 1952 |
| NWD340293 | dbGAP-CV | 1.659268 | 150376 | 137244 | 137165 | 79 | 2.327955 | 13132 | 2005 |
| NWD343999 | dbGAP-CV | 1.646865 | 151399 | 138193 | 138188 | 5 | 2.319672 | 13206 | 1925 |
| NWD346393 | dbGAP-CV | 1.558757 | 148203 | 135135 | 135126 | 9 | 2.336609 | 13068 | 1916 |
| NWD346468 | dbGAP-CV | 1.612018 | 150439 | 137218 | 137117 | 101 | 2.322921 | 13221 | 1938 |
| NWD347779 | dbGAP-CV | 1.72591 | 156087 | 142274 | 142271 | 3 | 2.342991 | 13813 | 2047 |
| NWD349716 | dbGAP-CV | 1.603545 | 149544 | 136269 | 136176 | 93 | 2.335031 | 13275 | 1915 |
| NWD352933 | dbGAP-CV | 1.490815 | 148130 | 135129 | 135042 | 87 | 2.32911 | 13001 | 1931 |
| NWD353514 | dbGAP-CV | 1.610983 | 149053 | 135917 | 135915 | 2 | 2.327335 | 13136 | 1908 |
| NWD361784 | dbGAP-CV | 1.745699 | 152930 | 139476 | 139355 | 121 | 2.325657 | 13454 | 1984 |
| NWD365731 | dbGAP-CV | 1.610807 | 149429 | 136220 | 136173 | 47 | 2.342653 | 13209 | 1964 |
| NWD366029 | dbGAP-CV | 1.602047 | 147941 | 134915 | 134915 | 0 | 2.334693 | 13026 | 1934 |
| NWD373460 | dbGAP-CV | 1.843823 | 156585 | 142664 | 142662 | 2 | 2.309946 | 13921 | 2031 |
| NWD375551 | dbGAP-CV | 1.709964 | 152012 | 138737 | 138712 | 25 | 2.334102 | 13275 | 1946 |
| NWD376234 | dbGAP-CV | 1.670677 | 149388 | 136166 | 136140 | 26 | 2.318626 | 13222 | 1941 |
| NWD381620 | dbGAP-CV | 1.559075 | 147350 | 134417 | 134387 | 30 | 2.327976 | 12933 | 1985 |
| NWD383240 | dbGAP-CV | 1.656169 | 152791 | 139419 | 139135 | 284 | 2.334572 | 13372 | 1956 |
| NWD383901 | dbGAP-CV | 1.743707 | 152115 | 138666 | 138666 | 0 | 2.325563 | 13449 | 1968 |
| NWD386997 | dbGAP-CV | 1.670273 | 151297 | 138033 | 138017 | 16 | 2.337775 | 13264 | 1980 |
| NWD388907 | dbGAP-CV | 1.695713 | 152462 | 139005 | 138967 | 38 | 2.34216 | 13457 | 1978 |
| NWD396722 | dbGAP-CV | 1.666281 | 149831 | 136633 | 136591 | 42 | 2.336289 | 13198 | 1926 |
| NWD401180 | dbGAP-CV | 1.729146 | 151987 | 138483 | 138393 | 90 | 2.332603 | 13504 | 1969 |
| NWD401962 | dbGAP-CV | 1.6453 | 148995 | 135967 | 135966 | 1 | 2.320861 | 13028 | 1952 |
| NWD407487 | dbGAP-CV | 1.635425 | 150644 | 137551 | 137465 | 86 | 2.339123 | 13093 | 1948 |
| NWD411668 | dbGAP-CV | 1.677858 | 151314 | 137962 | 137913 | 49 | 2.341482 | 13352 | 1950 |
| NWD415466 | dbGAP-CV | 1.647797 | 149759 | 136636 | 136636 | 0 | 2.334781 | 13123 | 1934 |
| NWD420735 | dbGAP-CV | 1.762342 | 153239 | 139800 | 139800 | 0 | 2.340821 | 13439 | 1967 |
| NWD421513 | dbGAP-CV | 1.6255 | 149269 | 136356 | 136345 | 11 | 2.336147 | 12913 | 1886 |
| NWD423586 | dbGAP-CV | 1.6785 | 150995 | 137796 | 137792 | 4 | 2.329516 | 13199 | 1946 |
| NWD426865 | dbGAP-CV | 1.53341 | 148958 | 135791 | 135790 | 1 | 2.321592 | 13167 | 1925 |
| NWD428764 | dbGAP-CV | 1.560765 | 148114 | 134954 | 134874 | 80 | 2.35149 | 13160 | 1908 |
| NWD429349 | dbGAP-CV | 1.572815 | 148751 | 135638 | 135623 | 15 | 2.336113 | 13113 | 1936 |
| NWD430931 | dbGAP-CV | 1.612235 | 149459 | 136202 | 136200 | 2 | 2.315805 | 13257 | 1920 |
| NWD435635 | dbGAP-CV | 1.54997 | 148181 | 135050 | 135050 | 0 | 2.336463 | 13131 | 1873 |
| NWD437315 | dbGAP-CV | 1.69943 | 150458 | 137334 | 137285 | 49 | 2.324092 | 13124 | 1913 |
| NWD439370 | dbGAP-CV | 1.622669 | 148393 | 135330 | 135288 | 42 | 2.336326 | 13063 | 1948 |
| NWD443983 | dbGAP-CV | 1.698512 | 152932 | 139426 | 139299 | 127 | 2.333469 | 13506 | 1957 |
| NWD445187 | dbGAP-CV | 1.692382 | 150992 | 137734 | 137649 | 85 | 2.32791 | 13258 | 1942 |
| NWD449443 | dbGAP-CV | 1.625004 | 150408 | 137232 | 137188 | 44 | 2.331828 | 13176 | 1925 |
| NWD451591 | dbGAP-CV | 1.57755 | 148688 | 135621 | 135617 | 4 | 2.331786 | 13067 | 1975 |
| NWD455939 | dbGAP-CV | 1.869147 | 159458 | 145402 | 145174 | 228 | 2.342635 | 14056 | 2040 |
| NWD469263 | dbGAP-CV | 1.72187 | 151187 | 137854 | 137809 | 45 | 2.32639 | 13333 | 2018 |
| NWD478360 | dbGAP-CV | 1.646411 | 151349 | 137998 | 137894 | 104 | 2.336011 | 13351 | 1955 |
| NWD480155 | dbGAP-CV | 1.754561 | 154210 | 140715 | 140665 | 50 | 2.327302 | 13495 | 2079 |
| NWD489083 | dbGAP-CV | 1.666617 | 149929 | 136708 | 136702 | 6 | 2.327702 | 13221 | 1898 |
| NWD491083 | dbGAP-CV | 1.742573 | 151285 | 137915 | 137866 | 49 | 2.323273 | 13370 | 1946 |
| NWD497224 | dbGAP-CV | 1.603552 | 149675 | 136698 | 136683 | 15 | 2.336906 | 12977 | 1841 |
| NWD500480 | dbGAP-CV | 1.636048 | 150816 | 137520 | 137518 | 2 | 2.331993 | 13296 | 1988 |
| NWD502650 | dbGAP-CV | 1.676163 | 149768 | 136595 | 136564 | 31 | 2.323048 | 13173 | 1928 |
| NWD503184 | dbGAP-CV | 1.686305 | 150324 | 137129 | 137123 | 6 | 2.337138 | 13195 | 1918 |
| NWD511161 | dbGAP-CV | 1.713954 | 150801 | 137560 | 137522 | 38 | 2.327091 | 13241 | 1932 |
| NWD519898 | dbGAP-CV | 1.61251 | 150550 | 137404 | 137392 | 12 | 2.329908 | 13146 | 1900 |
| NWD525158 | dbGAP-CV | 1.615434 | 151556 | 138168 | 138111 | 57 | 2.342473 | 13388 | 1947 |
| NWD528909 | dbGAP-CV | 1.693514 | 152994 | 139491 | 139340 | 151 | 2.326093 | 13503 | 1978 |
| NWD529614 | dbGAP-CV | 1.765645 | 155018 | 141406 | 141402 | 4 | 2.330554 | 13612 | 2038 |
| NWD532780 | dbGAP-CV | 1.777708 | 152767 | 139471 | 139348 | 123 | 2.32986 | 13296 | 1907 |
| NWD533352 | dbGAP-CV | 1.667122 | 152197 | 138675 | 138624 | 51 | 2.328307 | 13522 | 2008 |
| NWD533424 | dbGAP-CV | 1.752902 | 152403 | 138946 | 138887 | 59 | 2.309591 | 13457 | 1980 |
| NWD542232 | dbGAP-CV | 1.743041 | 155303 | 141523 | 141441 | 82 | 2.324269 | 13780 | 2033 |
| NWD549645 | dbGAP-CV | 1.598575 | 148478 | 135331 | 135331 | 0 | 2.32713 | 13147 | 1923 |
| NWD553845 | dbGAP-CV | 1.655719 | 149544 | 136418 | 136414 | 4 | 2.325711 | 13126 | 1993 |
| NWD556152 | dbGAP-CV | 1.658211 | 149596 | 136481 | 136385 | 96 | 2.342606 | 13115 | 1939 |
| NWD568742 | dbGAP-CV | 1.574399 | 148342 | 135272 | 135263 | 9 | 2.322925 | 13070 | 1938 |
| NWD572136 | dbGAP-CV | 1.656508 | 149317 | 136224 | 136207 | 17 | 2.329024 | 13093 | 1955 |
| NWD572300 | dbGAP-CV | 1.631626 | 149651 | 136624 | 136618 | 6 | 2.332797 | 13027 | 1892 |
| NWD575181 | dbGAP-CV | 1.673903 | 150700 | 137377 | 137376 | 1 | 2.338258 | 13323 | 1963 |
| NWD576325 | dbGAP-CV | 1.724731 | 153753 | 140212 | 140124 | 88 | 2.332873 | 13541 | 1983 |
| NWD592749 | dbGAP-CV | 1.721343 | 151452 | 138122 | 137999 | 123 | 2.328806 | 13330 | 1958 |
| NWD593180 | dbGAP-CV | 1.775278 | 155049 | 141397 | 141394 | 3 | 2.328092 | 13652 | 2016 |
| NWD599946 | dbGAP-CV | 1.649689 | 149672 | 136475 | 136474 | 1 | 2.331153 | 13197 | 1970 |
| NWD607594 | dbGAP-CV | 1.672303 | 150500 | 137365 | 137358 | 7 | 2.324169 | 13135 | 1943 |
| NWD609869 | dbGAP-CV | 1.634953 | 151662 | 138115 | 138087 | 28 | 2.329965 | 13547 | 1984 |
| NWD614013 | dbGAP-CV | 1.622903 | 149793 | 136416 | 136365 | 51 | 2.343263 | 13377 | 1918 |
| NWD621181 | dbGAP-CV | 1.692501 | 154163 | 140562 | 140502 | 60 | 2.334251 | 13601 | 2035 |
| NWD627618 | dbGAP-CV | 1.679977 | 149319 | 136239 | 136160 | 79 | 2.346359 | 13080 | 1901 |
| NWD630361 | dbGAP-CV | 1.664759 | 150005 | 136738 | 136737 | 1 | 2.327177 | 13267 | 1981 |
| NWD633520 | dbGAP-CV | 1.654591 | 149476 | 136250 | 136242 | 8 | 2.331182 | 13226 | 1936 |
| NWD633757 | dbGAP-CV | 1.535369 | 149538 | 136403 | 136403 | 0 | 2.324065 | 13135 | 1978 |
| NWD635742 | dbGAP-CV | 1.702126 | 151547 | 138100 | 138055 | 45 | 2.339986 | 13447 | 1958 |
| NWD639450 | dbGAP-CV | 1.649597 | 148947 | 135788 | 135787 | 1 | 2.335061 | 13159 | 1932 |
| NWD644576 | dbGAP-CV | 1.773296 | 156738 | 142840 | 142838 | 2 | 2.330877 | 13898 | 2036 |
| NWD647073 | dbGAP-CV | 1.579934 | 148587 | 135424 | 135414 | 10 | 2.332611 | 13163 | 1899 |
| NWD652119 | dbGAP-CV | 1.659898 | 149307 | 136165 | 136113 | 52 | 2.325507 | 13142 | 1927 |
| NWD655155 | dbGAP-CV | 1.69971 | 150598 | 137261 | 137182 | 79 | 2.330873 | 13337 | 1962 |
| NWD657109 | dbGAP-CV | 1.732234 | 151330 | 138046 | 137961 | 85 | 2.312548 | 13284 | 1979 |
| NWD658520 | dbGAP-CV | 1.63706 | 150026 | 136708 | 136708 | 0 | 2.337109 | 13318 | 1927 |
| NWD662457 | dbGAP-CV | 1.670677 | 150256 | 136996 | 136931 | 65 | 2.333763 | 13260 | 1955 |
| NWD668871 | dbGAP-CV | 1.658802 | 150955 | 137576 | 137575 | 1 | 2.334747 | 13379 | 1924 |
| NWD673356 | dbGAP-CV | 1.691026 | 149811 | 136575 | 136574 | 1 | 2.329611 | 13236 | 1901 |
| NWD678458 | dbGAP-CV | 1.637451 | 151267 | 138001 | 137936 | 65 | 2.328411 | 13266 | 1947 |
| NWD678602 | dbGAP-CV | 1.683455 | 151159 | 137944 | 137865 | 79 | 2.330153 | 13215 | 1953 |
| NWD678712 | dbGAP-CV | 1.383859 | 146281 | 133479 | 133429 | 50 | 2.321691 | 12802 | 1916 |
| NWD683837 | dbGAP-CV | 1.681482 | 150675 | 137499 | 137489 | 10 | 2.337436 | 13176 | 1973 |
| NWD684446 | dbGAP-CV | 1.627429 | 150725 | 137530 | 137504 | 26 | 2.335856 | 13195 | 1966 |
| NWD687697 | dbGAP-CV | 1.61751 | 151047 | 137632 | 137539 | 93 | 2.331936 | 13415 | 1949 |
| NWD688209 | dbGAP-CV | 1.706389 | 150793 | 137435 | 137408 | 27 | 2.334094 | 13358 | 1986 |
| NWD694068 | dbGAP-CV | 1.649104 | 149822 | 136637 | 136548 | 89 | 2.322578 | 13185 | 1921 |
| NWD695785 | dbGAP-CV | 1.718481 | 151382 | 138027 | 138026 | 1 | 2.327451 | 13355 | 1913 |
| NWD695880 | dbGAP-CV | 1.711278 | 153588 | 140038 | 139990 | 48 | 2.33135 | 13550 | 1961 |
| NWD697885 | dbGAP-CV | 1.65964 | 149533 | 136305 | 136264 | 41 | 2.338331 | 13228 | 1935 |
| NWD705178 | dbGAP-CV | 1.649791 | 149854 | 136824 | 136813 | 11 | 2.33008 | 13030 | 1930 |
| NWD707533 | dbGAP-CV | 1.68916 | 150535 | 137218 | 137165 | 53 | 2.328036 | 13317 | 1942 |
| NWD714294 | dbGAP-CV | 1.715763 | 153263 | 139887 | 139875 | 12 | 2.322368 | 13376 | 1934 |
| NWD723093 | dbGAP-CV | 1.759139 | 154668 | 141204 | 141049 | 155 | 2.32702 | 13464 | 1995 |
| NWD725761 | dbGAP-CV | 1.683493 | 149661 | 136456 | 136454 | 2 | 2.315451 | 13205 | 1932 |
| NWD731524 | dbGAP-CV | 1.621129 | 149909 | 136622 | 136542 | 80 | 2.329805 | 13287 | 1944 |
| NWD734527 | dbGAP-CV | 1.657232 | 150151 | 136922 | 136882 | 40 | 2.310326 | 13229 | 1964 |
| NWD741589 | dbGAP-CV | 1.691579 | 152437 | 139032 | 139032 | 0 | 2.308159 | 13405 | 1977 |
| NWD742087 | dbGAP-CV | 1.611953 | 148985 | 135845 | 135769 | 76 | 2.343405 | 13140 | 1959 |
| NWD742772 | dbGAP-CV | 1.65184 | 151133 | 137828 | 137824 | 4 | 2.316904 | 13305 | 1982 |
| NWD764752 | dbGAP-CV | 2.000154 | 163215 | 148755 | 148588 | 167 | 2.325455 | 14460 | 2064 |
| NWD767610 | dbGAP-CV | 1.707405 | 150471 | 137270 | 137168 | 102 | 2.327624 | 13201 | 1963 |
| NWD770924 | dbGAP-CV | 1.611972 | 149559 | 136394 | 136327 | 67 | 2.329759 | 13165 | 1900 |
| NWD771767 | dbGAP-CV | 1.698873 | 151069 | 137734 | 137656 | 78 | 2.331865 | 13335 | 1950 |
| NWD786967 | dbGAP-CV | 1.677892 | 151139 | 137786 | 137698 | 88 | 2.318504 | 13353 | 1979 |
| NWD788119 | dbGAP-CV | 1.631541 | 150825 | 137530 | 137450 | 80 | 2.324706 | 13295 | 1981 |
| NWD797364 | dbGAP-CV | 1.645089 | 149686 | 136378 | 136377 | 1 | 2.329842 | 13308 | 1933 |
| NWD797498 | dbGAP-CV | 1.718034 | 151229 | 137822 | 137722 | 100 | 2.309034 | 13407 | 1948 |
| NWD805292 | dbGAP-CV | 1.627719 | 149613 | 136413 | 136413 | 0 | 2.319536 | 13200 | 1902 |
| NWD807987 | dbGAP-CV | 1.70676 | 150892 | 137479 | 137397 | 82 | 2.318608 | 13413 | 1916 |
| NWD809272 | dbGAP-CV | 1.778814 | 157469 | 143420 | 143290 | 130 | 2.323514 | 14049 | 2013 |
| NWD811284 | dbGAP-CV | 1.699309 | 151701 | 138296 | 138214 | 82 | 2.31512 | 13405 | 1951 |
| NWD822216 | dbGAP-CV | 1.703378 | 151024 | 137717 | 137633 | 84 | 2.333245 | 13307 | 1962 |
| NWD826645 | dbGAP-CV | 1.573889 | 148505 | 135321 | 135259 | 62 | 2.327405 | 13184 | 1912 |
| NWD834686 | dbGAP-CV | 1.765077 | 158256 | 144275 | 144251 | 24 | 2.329894 | 13981 | 2003 |
| NWD842737 | dbGAP-CV | 1.726455 | 149294 | 136295 | 136278 | 17 | 2.321181 | 12999 | 1942 |
| NWD849563 | dbGAP-CV | 1.755877 | 150627 | 137407 | 137378 | 29 | 2.335551 | 13220 | 1903 |
| NWD849612 | dbGAP-CV | 1.637248 | 151584 | 138248 | 138158 | 90 | 2.325422 | 13336 | 2005 |
| NWD851056 | dbGAP-CV | 1.584398 | 147134 | 134139 | 134108 | 31 | 2.328898 | 12995 | 1899 |
| NWD861737 | dbGAP-CV | 1.787496 | 152256 | 138872 | 138751 | 121 | 2.327761 | 13384 | 1990 |
| NWD861942 | dbGAP-CV | 1.622635 | 149341 | 136129 | 136018 | 111 | 2.33852 | 13212 | 1983 |
| NWD867285 | dbGAP-CV | 1.598434 | 150670 | 137270 | 137204 | 66 | 2.333673 | 13400 | 1984 |
| NWD869246 | dbGAP-CV | 1.64016 | 150715 | 137461 | 137419 | 42 | 2.315936 | 13254 | 1976 |
| NWD873855 | dbGAP-CV | 1.653369 | 150617 | 137503 | 137501 | 2 | 2.316394 | 13114 | 1953 |
| NWD874486 | dbGAP-CV | 1.59957 | 149241 | 136114 | 136077 | 37 | 2.349835 | 13127 | 1906 |
| NWD880377 | dbGAP-CV | 1.654367 | 152046 | 138726 | 138622 | 104 | 2.326901 | 13320 | 1948 |
| NWD882738 | dbGAP-CV | 1.69612 | 149736 | 136533 | 136479 | 54 | 2.327133 | 13203 | 1898 |
| NWD889292 | dbGAP-CV | 1.485871 | 147434 | 134383 | 134318 | 65 | 2.328163 | 13051 | 1882 |
| NWD889765 | dbGAP-CV | 1.665199 | 150594 | 137359 | 137284 | 75 | 2.334969 | 13235 | 1969 |
| NWD893155 | dbGAP-CV | 1.714835 | 150146 | 136972 | 136970 | 2 | 2.336663 | 13174 | 1934 |
| NWD894617 | dbGAP-CV | 1.81122 | 157524 | 143675 | 143657 | 18 | 2.331872 | 13849 | 2104 |
| NWD895913 | dbGAP-CV | 1.591982 | 147873 | 134940 | 134627 | 313 | 2.331939 | 12933 | 1980 |
| NWD897943 | dbGAP-CV | 1.711821 | 150301 | 137122 | 137122 | 0 | 2.337357 | 13179 | 1946 |
| NWD902021 | dbGAP-CV | 1.63729 | 151266 | 137879 | 137824 | 55 | 2.332785 | 13387 | 1973 |
| NWD905248 | dbGAP-CV | 1.619281 | 149971 | 136685 | 136641 | 44 | 2.332138 | 13286 | 1950 |
| NWD913101 | dbGAP-CV | 1.665282 | 148821 | 135750 | 135695 | 55 | 2.322844 | 13071 | 1928 |
| NWD918403 | dbGAP-CV | 1.547626 | 148182 | 135116 | 135049 | 67 | 2.326986 | 13066 | 1897 |
| NWD929473 | dbGAP-CV | 1.739125 | 152524 | 139049 | 138960 | 89 | 2.325914 | 13475 | 1964 |
| NWD933744 | dbGAP-CV | 1.672636 | 151533 | 138123 | 138017 | 106 | 2.329803 | 13410 | 2000 |
| NWD940790 | dbGAP-CV | 1.700477 | 150625 | 137444 | 137371 | 73 | 2.327625 | 13181 | 1952 |
| NWD942200 | dbGAP-CV | 1.656897 | 149020 | 135989 | 135956 | 33 | 2.31859 | 13031 | 1956 |
| NWD943005 | dbGAP-CV | 1.626204 | 150497 | 137354 | 137350 | 4 | 2.338925 | 13143 | 1940 |
| NWD945441 | dbGAP-CV | 1.601075 | 149708 | 136600 | 136597 | 3 | 2.327819 | 13108 | 1885 |
| NWD946043 | dbGAP-CV | 1.713122 | 150087 | 136729 | 136671 | 58 | 2.351094 | 13358 | 1961 |
| NWD955901 | dbGAP-CV | 1.689259 | 151179 | 137904 | 137826 | 78 | 2.336787 | 13275 | 1952 |
| NWD966349 | dbGAP-CV | 1.666086 | 149472 | 136293 | 136242 | 51 | 2.321113 | 13179 | 1935 |
| NWD969419 | dbGAP-CV | 1.571525 | 148914 | 135866 | 135802 | 64 | 2.322373 | 13048 | 1909 |
| NWD971027 | dbGAP-CV | 1.692485 | 150727 | 137595 | 137592 | 3 | 2.321793 | 13132 | 1910 |
| NWD973867 | dbGAP-CV | 1.631667 | 149470 | 136321 | 136283 | 38 | 2.330637 | 13149 | 1955 |
| NWD978003 | dbGAP-CV | 1.47597 | 148762 | 135599 | 135524 | 75 | 2.338852 | 13163 | 1938 |
| NWD981775 | dbGAP-CV | 1.597646 | 150668 | 137720 | 137697 | 23 | 2.331486 | 12948 | 1905 |
| NWD990575 | dbGAP-CV | 1.592925 | 147904 | 134939 | 134939 | 0 | 2.333473 | 12965 | 1913 |
| NWD994356 | dbGAP-CV | 1.652961 | 149502 | 136346 | 136327 | 19 | 2.333994 | 13156 | 1936 |
| NWD994436 | dbGAP-CV | 1.673038 | 149720 | 136565 | 136534 | 31 | 2.337342 | 13155 | 1931 |
| NWD996801 | dbGAP-CV | 1.686155 | 151180 | 137850 | 137847 | 3 | 2.34045 | 13330 | 1969 |

**Supplementary Table 3.** Mean ancestry component of each population used in this study using 446 and 78 AIMS, from Galanter et al (2012) and Campos-Sánchez et al. (2013), respectively. Ancestral populations are 200 individuals from 1KGP: AFR - Africans, EAS - East Asians, and EUR - Europeans. CLM: Colombia, MXL: Mexico, PEL: Peru, PUR: Puerto Rico, PSYCH-CV: Psychiatric study Central Valley, dbGAP-CV: dbGAP Central Valley, 2013-CR: Costa Ricans from the Caribbean Region, 2013-NR: Costa Ricans from the North Zone, 2013-SR: Costa Ricans from the South Zone, 2013-CV: Costa Ricans from the Central Valley.

|  |  | **446 AIMS** | | | **78 AIMS** | | |
| --- | --- | --- | --- | --- | --- | --- | --- |
| **Population** | **Sample size** | **AFR** | **EAS** | **EUR** | **AFR** | **EAS** | **EUR** |
| PSYCH-CV | 23 | 0.039 | 0.460 | 0.501 | 0.053 | 0.458 | 0.489 |
| dbGAP-CV | 234 | 0.032 | 0.412 | 0.556 | 0.043 | 0.470 | 0.487 |
| CLM | 94 | 0.075 | 0.368 | 0.557 |  |  |  |
| MLX | 64 | 0.017 | 0.637 | 0.346 |  |  |  |
| PEL | 85 | 0.018 | 0.898 | 0.084 |  |  |  |
| PUR | 104 | 0.137 | 0.193 | 0.670 |  |  |  |
| 2013-CR | 21 |  |  |  | 0.310 | 0.425 | 0.265 |
| 2013-NR | 37 |  |  |  | 0.147 | 0.586 | 0.267 |
| 2013-SR | 25 |  |  |  | 0.077 | 0.597 | 0.326 |
| 2013-CV | 77 |  |  |  | 0.056 | 0.525 | 0.419 |

**Supplementary Table 4.** Comparison of ancestry components using 95% confidence intervals in the populations of study. Data displayed as a difference (lower; upper bound). * p-value significant (>0.05)

| **Comparison** | **446 AIMS** | | | **78 AIMS** | | |
| --- | --- | --- | --- | --- | --- | --- |
|  | **AFR** | **EAS** | **EUR** | **AFR** | **EAS** | **EUR** |
| dbGAP-CV; PSYCH-CV | -0.022 (-0.157;0.114) | 0.032 (-0.076;0.14) | -0.052 (-0.134;0.031) | 0.017 (-0.152;0.186) | -0.018 (-0.146;0.11) | -0.003 (-0.126;0.12) |
| dbGAP-CV; CLM | -0.078 (-0.153;-0.002)* | 0.032 (-0.01;0.074) | 0.004 (-0.047;0.054) |  |  |  |
| dbGAP-CV; MLX | -0.122 (-0.209;-0.033)* | 0.135 (0.078;0.191)* | -0.174 (-0.25;-0.096)* |  |  |  |
| dbGAP-CV; PEL | -0.262 (-0.35;-0.17)* | 0.269 (0.236;0.303)* | -0.431 (-0.486;-0.373)* |  |  |  |
| dbGAP-CV; PUR | 0.227 (0.18;0.274)* | -0.158 (-0.185;-0.132)* | 0.088 (0.051;0.126)* |  |  |  |
| PSYCH-CV; CLM | -0.1 (-0.241;0.045) | 0.065 (-0.048;0.176) | -0.049 (-0.137;0.041) |  |  |  |
| PSYCH-CV; MLX | 0.1 (-0.053;0.248) | -0.102 (-0.213;0.011) | 0.122 (0.016;0.225)* |  |  |  |
| PSYCH-CV; PEL | 0.24 (0.091;0.379)* | -0.237 (-0.332;-0.137)* | 0.379 (0.293;0.458)* |  |  |  |
| PSYCH-CV; PUR | 0.249 (0.12;0.371)* | -0.191 (-0.296;-0.081)* | 0.141 (0.059;0.221)* |  |  |  |
| PSYCH-CV; 2013-CV |  |  |  | -0.063 (-0.242;0.121) | -0.087 (-0.224;0.054) | 0.069 (-0.066;0.202) |
| PSYCH-CV; 2013-CR |  |  |  | -0.242 (-0.46;0.003) | -0.005 (-0.201;0.191) | 0.256 (0.075;0.42)* |
| PSYCH-CV; 2013-SR |  |  |  | -0.088 (-0.308;0.14) | -0.198 (-0.342;-0.044)* | 0.192 (0.044;0.332)* |
| PSYCH-CV; 2013-NR |  |  |  | -0.268 (-0.445;-0.072)* | -0.177 (-0.32;-0.026)* | 0.253 (0.112;0.384)* |
| dbGAP-CV; 2013-CV |  |  |  | -0.08 (-0.174;0.016) | -0.068 (-0.143;0.008) | 0.072 (0;0.143)* |
| dbGAP-CV; 2013-CR |  |  |  | -0.259 (-0.431;-0.069)* | 0.013 (-0.147;0.174) | 0.259 (0.119;0.389)* |
| dbGAP-CV; 2013-SR |  |  |  | -0.106 (-0.269;0.063) | -0.179 (-0.274;-0.081)* | 0.196 (0.102;0.286)* |
| dbGAP-CV; 2013-NR |  |  |  | -0.286 (-0.396;-0.166)* | -0.158 (-0.251;-0.063)* | 0.256 (0.169;0.339)* |
